# Identification and Mapping of Linear Antibody Epitopes in Human Serum Albumin Using High-Density Peptide Arrays

**DOI:** 10.1371/journal.pone.0068902

**Published:** 2013-07-23

**Authors:** Lajla Bruntse Hansen, Soren Buus, Claus Schafer-Nielsen

**Affiliations:** 1 Laboratory of Experimental Immunology, University of Copenhagen, Copenhagen, Denmark; 2 Schafer-N, Copenhagen, Denmark; Technical University of Denmark, Denmark

## Abstract

We have recently developed a high-density photolithographic, peptide array technology with a theoretical upper limit of 2 million different peptides per array of 2 cm^2^. Here, we have used this to perform complete and exhaustive analyses of linear B cell epitopes of a medium sized protein target using human serum albumin (HSA) as an example. All possible overlapping 15-mers from HSA were synthesized and probed with a commercially available polyclonal rabbit anti-HSA antibody preparation. To allow for identification of even the weakest epitopes and at the same time perform a detailed characterization of key residues involved in antibody binding, the array also included complete single substitution scans (i.e. including each of the 20 common amino acids) at each position of each 15-mer peptide. As specificity controls, all possible 15-mer peptides from bovine serum albumin (BSA) and from rabbit serum albumin (RSA) were included as well. The resulting layout contained more than 200.000 peptide fields and could be synthesized in a single array on a microscope slide. More than 20 linear epitope candidates were identified and characterized at high resolution i.e. identifying which amino acids in which positions were needed, or not needed, for antibody interaction. As expected, moderate cross-reaction with some peptides in BSA was identified whereas no cross-reaction was observed with peptides from RSA. We conclude that high-density peptide microarrays are a very powerful methodology to identify and characterize linear antibody epitopes, and should advance detailed description of individual specificities at the single antibody level as well as serologic analysis at the proteome-wide level.

## Introduction

Ideally, the epitope(s) targeted by antibodies used as e.g. diagnostic or therapeutic tools should be identified and extensively characterized in order to validate specificity and to document cross-reactivity that otherwise might lead to spurious results. Unfortunately, current methods of physicochemical epitope characterization tend to be costly, cumbersome, and of low throughput. Examples include X-ray crystallography [1], [2] and multidimensional NMR [3], [4]. As golden standards of epitope characterization these methodologies allow precise identification of the amino acid side chains involved in binding, but they are not suited for large-scale epitope identification and their results cannot be interpreted readily in terms of possible cross-reactions. Other epitope mapping approaches include proteolytic fragmentation [5], analysis of protein arrays and peptide arrays [6], or analysis of recombinant antigen (including antigens arrayed by in situ cell-free translation [7], mutagenized [8] and/or expressed using selectable systems such as phage display [9]). Despite this plethora of epitope-mapping methods, detailed epitope information lacks for the vast majority of antibodies used in life science research. Thus, there is a significant need for comprehensive, yet simple and rapid, methods to map epitopes. Proteins constitute important immune targets and many antibodies used for therapeutic or diagnostic purposes are targeting protein antigens. Traditionally, antibody epitopes in proteins have been classified as being either conformational, i.e. being functional only in spatially constrained forms, or as being linear, i.e. being functional in a form that may be represented by unconstrained peptides [10], [11]. Libraries of linear peptides and of peptides with simple spatial constraints can be produced in various formats and have been used extensively in screenings of antibody epitopes. Early approaches to the synthesis of synthetic peptide libraries involved solid phase synthesis on polystyrene pins (Geysen et al. 1984 [12]), on beads contained in “tea-bags” (Houghten et al. 1985 [13]) or on beads that through a “mix-and-split” strategy (Furka et al. 1991 [14]) allowed the synthesis of “one-bead-one-peptide” libraries (Lam et al. 1991 [15]). While these techniques are able to generate large numbers of different peptides they either require complicated logistics for peptide tracking or they require post-assay sequencing of the positive peptides.

Peptide arrays synthesized and analyzed on planar surfaces simplify the logistics of handling large numbers of peptides and eliminate the need for identification of peptides by sequencing. In a seminal paper, Fodor et al. [16] described the generation of peptide microarrays using a semi-automatic light-directed chemical synthesis of multiple peptides on glass surfaces. Shortly after, Frank et al. [17] described a competing approach involving fully automated synthesis of arrays of peptides with predefined sequences on paper membranes (“SPOT® synthesis”) and this together with the pin-based “PepScan®” method [12] has become a preferred method of generating peptide microarrays. Using SPOT® synthesis, peptide microarray with up to 8000 peptides have been reported [18], and recently high-density peptide microarrays made using a special laser printer technology have been introduced [19]. Light-directed synthesis of peptides has been facilitated by replacement of the physical masks used by Fodor et al. with a digital mirror device (Singh-Gasson et al. [20]) and by introduction of strategies using amino acids with standard protection groups rather than photosensitive groups (Li et al. [21]). Along these lines, we have recently developed a peptide microarray technology that is capable of synthesizing peptide microarrays with up to 2 million pre-addressable peptide fields. Here, we have used this technology to make a complete mapping of linear antibody epitopes in a readily available average sized protein, human serum albumin (HSA) using commercially available polyclonal rabbit anti-HSA antibody as the probe. In a single peptide microarray featuring more than 200.000 peptide fields on a 2 cm^2^ area, we included 5 copies of all possible 15-mer peptides from HSA. For each of the 595 different 15-mers, we included a complete single-residue substitution analysis with each of the 20 common amino acids and as specificity controls we included all possible 15-mer peptides of bovine serum albumin (BSA) and of rabbit serum albumin (RSA). Using this integrated approach combined with a statistical analysis of the results, we suggest that rabbit anti-HSA antibodies recognize more than 20 linear epitopes and within each of these epitopes we identified the residue positions important for antibody binding together with the amino acid preference of these residues. As expected from the specifications of the manufacturer, the rabbit anti-HSA antibodies showed moderate cross-reactions towards peptides from BSA, while no reaction was observed toward peptides from RSA. We conclude that a systematic overlap and single-residue substitution analysis as enabled by the high-density peptide microarray technology described here allows simultaneous mapping of multiple linear B cell epitopes at the single-residue level.

## Methods

### Synthesis of High-density Peptide Arrays

Layouts of the arrays were made with proprietary software using the FASTA sequences of HSA (UniProt P02768 Isoform 1), BSA (UniProt P02769) and RSA (RefSeq NP_990592) as input together with definitions of the desired peptide lengths, overlaps, number of copies and amino acids used for single-residue substitutions.

A high-density peptide array was generated using maskless photolithographic synthesis [20] adapted to solid phase peptide synthesis with the C-terminal anchored to the surface [22], [23], [21]. Except for a change in the synthesis substrate (described below) the synthesis was performed as detailed by Schafer-Nielsen and coworkers [24]. Briefly, the image patterns were generated using a.95″ 1920×1080 digital mirror device (DMD, Digital Light Innovations, Austin, Texas) illuminated with collimated 365 nm UV-light. The image patterns generated by 10×10 µm mirrors on the DMD were projected onto the synthesis substrate using 1∶1 UV-imaging optics. The synthesis substrate was a HiSens E microscope slide (Schott AG, Germany) coated by overnight incubation with a 2% w/v linear copolymer of N,N′-dimethylacrylamide and aminoethyl methacrylate (both from Sigma-Aldrich) mixed in a 20∶1 w/w ratio before polymerization for 2 hours at room temperature in freshly degassed 0.1 M sodium borate buffer, pH 8 containing 0.025% v/v TEMED and 0.1% w/v ammonium persulfate**.** Coupling of amino acids was performed as in classical Fluorenylmethyloxycarbonyl chloride (Fmoc) peptide synthesis using standard Fmoc amino acids (0.1 M amino acid, 0.1 M O-Benzotriazole-N,N,N′,N′-tetramethyl-uronium-hexafluoro-phosphate (HBTU), 0.2 M DIEA in N-methylpyrrolidone (NMP) premixed for 5 minutes before loading into the flow cell). Couplings were performed for 5 minutes after loading of the activated amino acid. The principal deviation from the classical strategy was that the photolabile (2-(2-nitrophenyl)propyl oxycarbonyl (NPPOC) was coupled to amino groups on the surface of the substrate before onset of synthesis, and when all n-terminal amino groups in a given layer had been coupled and the Fmoc-groups had been removed with 20% v/v piperidine in NMP for 20 minutes. Couplings of NPPOC were made by incubation for 30 min with a 1% v/v solution of NPPOC-chloroformate (Sigma), 0.1 M DIEA in DCM/NMP 1∶4 v/v. Before coupling with a new amino acid, relevant areas of the synthesis substrate were irradiated in 0.1 M DIEA in NMP for 10 minutes with 365 nm UV-light at an energy density of 20 mW/cm^2^. All synthesis steps were performed at room temperature by a proprietary liquid handling robot connected to a locally constructed DMD-projection device equipped with a flow cell holding the synthesis substrates. After synthesis, side chain protecting groups of peptides in the array were cleaved by incubation in Trifluoroacetic acid (TFA): 1,2-ethanedithiol (EDT): Triisopropylsilane (TIPS):H_2_O, 92∶2:1∶5 v/v/v/v overnight at room temperature before washing in DCM and air drying.

### Binding, Staining and Recording of the Array

The microscope slide with the high-density peptide array was incubated for 2 hours in polyclonal rabbit anti-human HSA (A001, DAKO) diluted 1+100 in 0.15 M Tris (Trizma® base, Sigma-Aldrich)/acetate pH 8.0, 0.1% v/v Tween20 (dilution and washing buffer). After washing, the slide was incubated for 2 hours in a 1+1000 dilution of Cy3-conjugated goat anti-rabbit IgG (A10520, InVitrogen) followed by repeated washings. Finally, the array was visualized and recorded using a MVX10 fluorescence microscope (Olympus) equipped with a MX10 cooled camera connected to a computer with CellP© imaging software (Olympus).

### Analysis of the Array Images

Digital images of the array were scanned and analyzed by proprietary software. The results were stored in text files as TAB-delimited lists including the sequences in each peptide field and the average fluorescence intensity recorded in that field. Background correction was performed by subtraction of the intensity recorded in nearby “blank” fields (i.e. fields with no peptide, only linker residues). The signal in the outer 30% of the rim of each field was discarded to minimize carryover of signals between the fields.

### Statistical Analysis

A complete single-residue scan was performed for each 15-mer peptide from HSA, and the signal obtained for each substituted peptide was subsequently expressed in percentage to the signal of the corresponding native 15-mer peptide. The normalized values were arranged in position specific scoring matrices (PSSM) with 15 columns representing the positions of the native peptide and 20 rows representing the 20 naturally occurring amino acids (Figure S2). An arithmetic mean of the normalized values, µ, was calculated for each position. Each PSSM was subjected to an analysis of variance (ANOVA) followed by a post-hoc analysis using Tukey’s Honest Significance Difference (HSD). The HSD value was used to identify positions where µ differed significantly from 100% and thus, contribute to the selectivity of the antibody-peptide interaction at a chosen significance level. HSD is calculated according to the formula
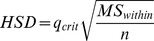
Where q_crit_ is the relevant critical value of the studentized range, i.e. the value found in q-tables corresponding to the available degrees of freedom at the chosen level of significance (p<0.01). *MS*_within_ is the mean square error and n is the number of substitutions used in calculation of the mean values.

The obtained value of the studentized range (q_obt_) is calculated as:
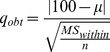



The obtained q-value is compared with the critical q-value by calculating the ratio (Rq) between these two values:
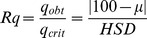



The ratio will be ≥1.0 for all positions with residues that contribute to the binding of antibody.

## Results

### Analysis of signals from a high-density array probed with anti-HSA antibodies

A microarray with more than 200.000 peptide fields was designed and synthesized as described in the method section. In addition to 15-mer peptides derived from the native sequences of HSA, BSA and RSA, the array contained 300 single-residue substituted variants of each of the 595 different 15-mers from HSA, and more than 20.000 reference peptide fields. The protein sequences of HSA, BSA and RSA are listed in Figure S1.

**Figure 1:**
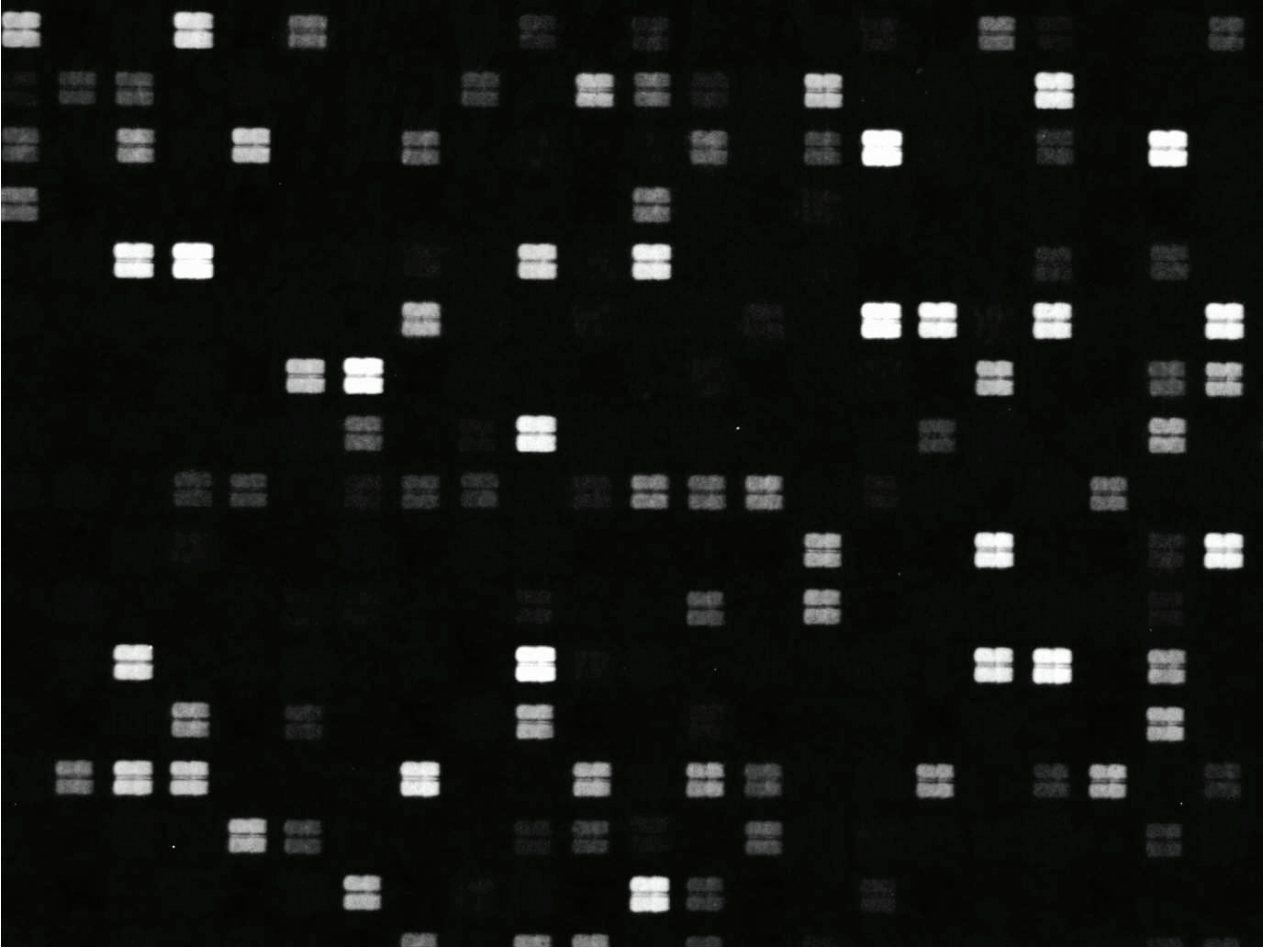
Image of array. A small section of the peptide array used for identification and fine specificity mapping of HSA epitopes: The section shows approximately 300 of the total 220.428 peptide fields in this array. The peptides were synthesized in predefined, addressable fields generated by 2×2 mirrors on the DMD each measuring 10×10 µm resulting in peptide fields with the size 20×20 of µm. The peptide fields were spaced by 10 µm wide empty zones. Binding of polyclonal rabbit anti-HSA antibody to the fields was recorded by fluorescence microscopy after incubation with Cy3-conjugated goat anti-rabbit IgG.

**Figure 2:**
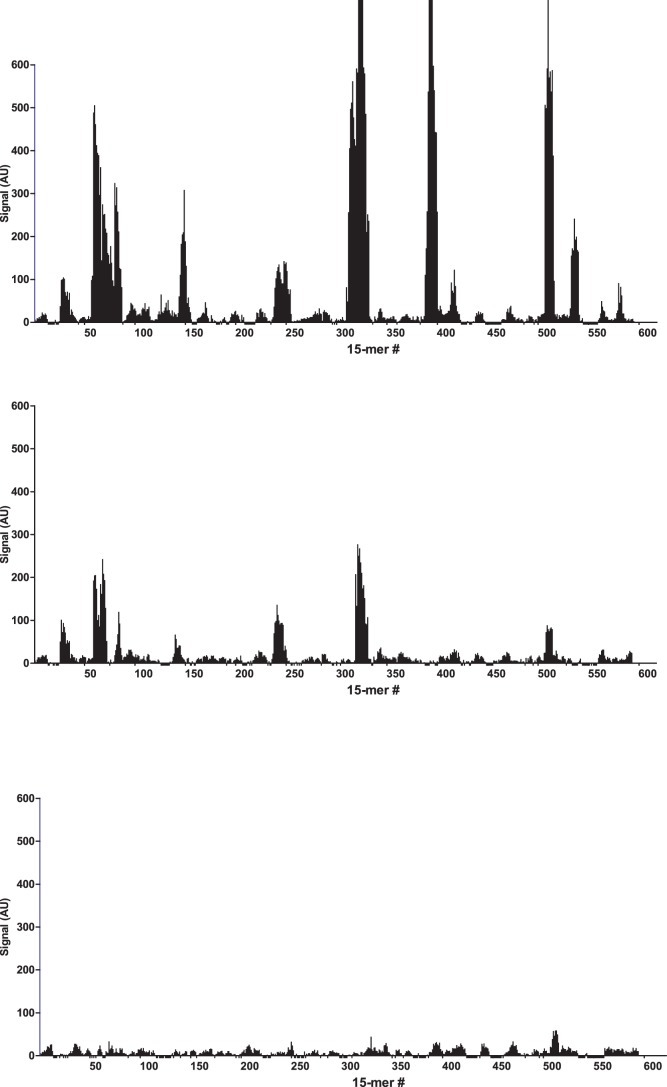
Signals. Bar chart displaying the fluorescence signal obtained from binding of polyclonal anti-HSA to 15-mer peptides with 14 residue overlap (average signal from 5 copies of each peptide). **A:** peptides from HSA, **B:** peptides from BSA and **C:** peptides from RSA. The peptides are numbered on the x-axis according to the position of their n-terminal residue in the protein sequence. The y-axis denotes the average intensity of the signal (AU) after background subtraction.

After binding of the antibody, images of the array (Fig. 1) recorded using fluorescence microscopy revealed discrete fluorescence of sharply demarcated quadratic fields consistent with a successful optical projection of the digital mirrors onto the peptide microarray. A plot of the signal intensities obtained from the overlapping 15-mer peptides in HSA (Fig. 2A) shows clear accumulation of signals corresponding to discrete regions of the HSA sequence. Both the width and the height of the signal peaks differ from one region to the next and depending on the criteria used for inclusion it can be readily estimated that 10–20 signal peaks are discernible.

### Off-target Specificity of the Antibodies

We tested whether the rabbit anti-HSA antibody showed any cross-reactivity towards serum albumin from other species. In spite of strong conservation between serum albumins from various species a certain amount of cross-reactivity might be expected. The manufacturer of the antibody has observed cross-reaction with xenogeneic bovine serum albumin (BSA) in rocket immunoelectrophoresis assays. In contrast, no cross-reactions would be expected with syngeneic rabbit serum albumin (RSA).

Overlapping 15-mer peptides from BSA and RSA were synthesized in 5 copies on the same peptide array, and detected with the rabbit anti-HSA antibody. Compared to HSA peptides (Fig. 2A), distinct, albeit weaker, signals were observed against peptides from BSA (Figure 2B). These BSA epitopes were homologous to the HSA epitopes seen in Figure 2A. In contrast, no reactions were observed against RSA peptides (Figure 2C). In all the identified epitopes from HSA, at least one of the amino acids critical for binding (as described below) differs from the corresponding residue in RSA (Figure S1). This, however, cannot solely explain the specificity of the rabbit anti-HSA antibody since similar differences are also observed in the regions where no epitopes candidates were found.

No signals were detected in a peptide microarray with the same overlapping HSA peptides probed with either polyclonal antibody from rabbits that had not been immunized with HSA or with only the secondary goat anti-rabbit IgG antibody (data not shown). In terms of reproducibility, the experiment was repeated leading to the identification of the same epitope regions (data uploaded to Dryad Data doi:10.5061/dryad.3003f). Using these specificity controls we conclude that the observed antibody reactions are exquisitely specific.

### Single-residue Substitution Analysis

By mere inspection of the signals in Figure 2A it cannot be determined if a small peak with a low signal actually represents an epitope. Neither can it be determined if a wide peak actually represents 2 or more adjacent epitopes. Therefore, a more objective analysis is warranted to identify, delineate and characterize each epitope candidate found in this experiment. Single-residue substitution with several amino acids is a very efficient tool in mapping of epitope sequences [12]. Thus, to elucidate the number of epitopes and at the same time identify the residue positions that are important for binding of antibody, a complete single-residue substitution analysis was performed for each of the 595 overlapping 15-mer peptides derived from HSA. A position specific scoring matrix (PSSM) was constructed for each 15-mer peptide (exemplified in Fig. S2). The PSSM contains a column for each amino acid position in the native peptide and a row for each amino acid used for single-residue substitution. Since we used 15-mer peptides and made single-residue substitutions with each of the 20 common amino acids, a PSSM contained 15×20 = 300 fields each displaying the signal obtained with a mono-substituted variant of the original 15-mer peptide. For convenience, the signals in the PSSM are expressed as percentages of the signal obtained with the native sequence. If a given residue position within the peptide is unimportant for binding of the peptide to the antibody, i.e. if the amino acid residues in the native sequence can be freely substituted without affecting binding, then the mean signal obtained at this non-selective position will be 100%. Positions with mean signals deviating significantly from 100% will accordingly be identified as being significant for binding of the antibody to the native peptide. In most cases substitution of the native residue in a significant position will result in attenuation of the signal, and in these cases the mean signal will be less than 100%. In the case that higher signals are obtained when the native residue is substituted with other amino acids - a situation known as a heteroclitic response [25] - then the mean signal can exceed 100%. These cases will not be further described or discussed here.

### Epitope Identification

**Figure 3:**
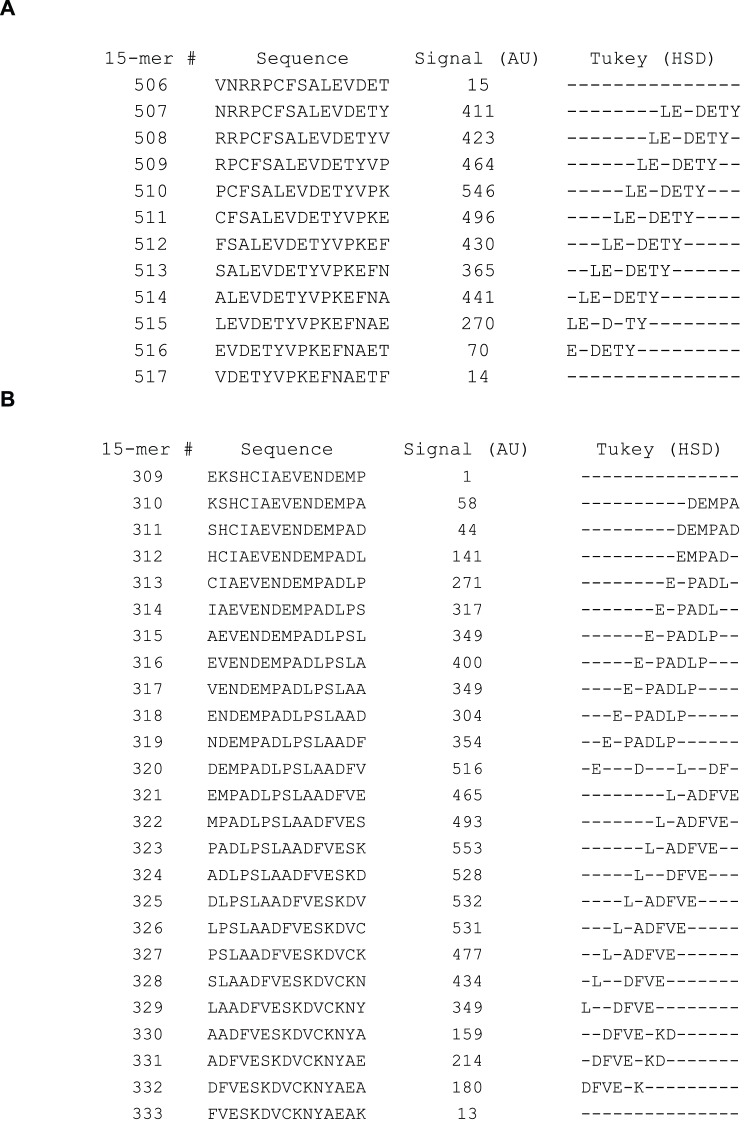
Example of Tukey’s HSD. The figure shows two examples example of epitope sequences identified using ANOVA followed by Tukey’s HSD post-hoc analysis. The leftmost column shows overlapping 15-mer peptides from HSA from position 510–527 in figure 3A and from position 309 to 333 in figure 3b. The rightmost column highlights the amino acids identified as being important for antibody binding (dashes indicate non-important positions). The important amino acids were determined on the p<0.01 level in the HSD post-hoc analysis.

To determine if the mean signal within a particular position deviates significantly from 100%, a one-way ANOVA analysis was performed for each of the 595 PSSM’s. The F-value calculated during the ANOVA is traditionally used to determine if the calculated variances are distributed randomly (our null hypothesis). With the large amount of data used in each PSSM, significant deviations (p<0.01, F>2. 04 for a given 15-mer individually) were found in 90% of the peptides. Considering that 595 F-tests were performed, we corrected for multiple testing and applied a threshold of F>3.54 corresponding to a 1% risk of one of 595 tests representing a type 1 error (p<1.689×10^−5^, F>3.54) (Table S1). When correcting for multiple testing, 78% of the peptides showed significant deviation from the null hypothesis. Due to the large number ANOVA positive PSSM’s (F>3.54) we performed Tukey’s HSD post hoc analysis for all columns in each PSSM irrespective of the calculated F-values. The HSD-value indicates how much the observed normalized mean of a given position must differ from 100% before the deviation is considered significant at the chosen level of significance (*in casu* p<0.01). Significant positions were found in approximately 18 peptide regions in HSA, which is exemplified in Figure 3a showing an epitope region sliding through peptide 507–516. In at least 4 of these regions two or more different patterns of significant positions were observed to slide through the region suggesting the existence of several different adjacent epitopes. This is exemplified in Figure 3b by peptides 309–333, and all the significant positions are given in its entirety in Table S1. It is not possible to determine whether the long stretches identified using a polyclonal antibody is bound by more than one antibody clone. Thus, it cannot be decided whether these regions contain one or more epitopes and no exact conclusion about the total number of epitopes recognized by our chosen polyclonal HSA-antibody are made. About 190 “epitope-significant” residue positions were found in HSA corresponding to about one-third of the amino acids in the mature form of HSA.

### Interactions Assigned at the Single-residue Level

Tukey’s HSD post-hoc analysis identified positions where substitutions significantly affected binding of antibody, but it did not quantitate the relative significance of the different residues. As described in the methods section, a measure of the relative significance was obtained by calculating the ratio between the obtained q-value (q_obt_) and the critical q-value (q_crit_). This ratio, Rq, can be calculated for each individual position in each peptide, and it describes the deviation of the signal in the position from 100% in units of significant differences (HSD).

**Figure 4:**
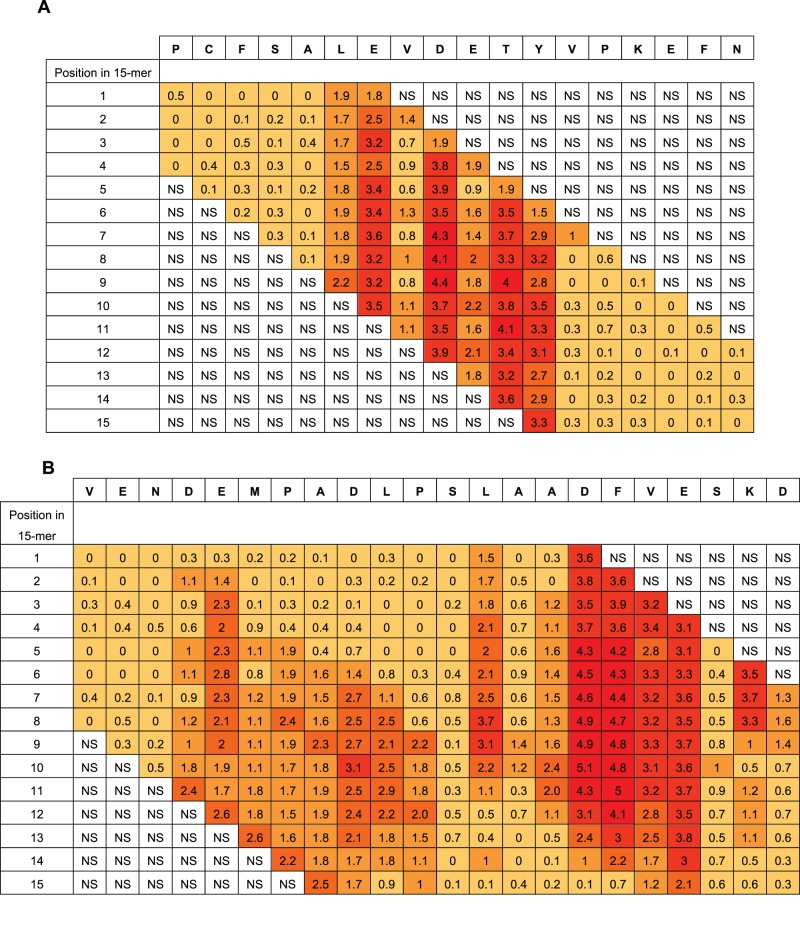
Example of mean Rq-ratio. The figure shows two examples using the Rq-values. Figure 4a shows residue 510–527 in the HSA sequence (top row) a sequence containing the epitope important residues LEVDETYV identified by Tukey’s HSD. Figure 4b shows residue 317 to residue 338 containing the epitope important residues DEMPADLP-LAADFVESKD. The column below each HSA residue lists the Rq-values calculated in each of the 15 overlapping peptides in which the residue is represented. Rq values from peptides with no positions identified by Tukey’s HSD test are set as non significant (NS) in the figure. The color indicates the size of the Rq value; darker color indicates higher Rq values.

In the extensive substitution analysis employed here, each position in the original HSA sequence was represented in each of 15 different peptides with different reading frames. The 15 Rq-values of a given residue differs with its position in a 15-mer peptide. However, when looking at given residue in all positions in the different 15-mers, a good statistical measure is obtained of the contribution of the residue compared to the other residues in the same epitope region. Two examples of the epitope Rq-values are seen in Figure 4a and 4b (all Rq-values are listed in Table S1). Since the Rq-value is expressed in units of significant differences (HSD), we chose only to look at the Rq from peptides containing residues that contribute significantly to binding of antibody as judged by Tukey’s post hoc analysis. The other peptides are judged as non-significant (NS). Figure 4a shows Rq values from position 510–527. The residues LE-DETY have been recognized by Tukey’s HSD (Figure 3a), and a measure of the contribution of the single residues are judged by the Rq-values. Figure 4b shows Rq values from position 317–338. The residues DEMPADLP-L-ADFVE-KD have been recognized by Tukey’s HSD (Figure 3b). Again, the contribution of the residues as judged by the Rq-values indicate a separation of the residues into two different epitopes with the last part of epitope (the second epitope) contributing more to the binding than the first part (keeping in mind that all Rq values above 1 indicates that the residue contributes to binding at a significance level below 0.01). Judging from the Rq values some of the larger epitope areas seems likely to be more than one epitopes and we suggest the existence of more than 20 HSA linear epitope candidates, although no exact conclusion can be made when polyclonal antibodies are used as probes. The epitope important residues are emphasized in boldface letters in the HSA sequence in Figure S1 and the epitope candidates are underlined.

## Discussion

Many different approaches to epitope mapping have been suggested over the years but they tend to be resource-intensive and have rather low throughput. Synthetic peptides have often been used in mapping of linear epitopes, but even modest peptide-driven epitope discovery programs can be severely strained by the costs and logistics of acquiring and handling large number of peptides. To overcome these disadvantages, we have recently implemented a new high-density peptide microarray technology and used it to perform exhaustive analysis of linear epitopes. To exemplify the power of this technology we have here extended this analysis to a model protein antigen in its entirety. To this end we have identified and fine-mapped multiple linear epitopes from a medium sized protein, HSA, as recognized by a polyclonal rabbit-anti HSA antibody. A single array containing more than 200.000 peptide fields was synthesized using a photolithographic approach and the data recorded on a single array allowed the linear epitopes to be mapped at an unprecedented level of detail greatly exceeding past epitope mapping experiments using synthetic peptides. Every possible 15-mer peptide from HSA was synthesized in several copies and immediately yielded a picture of 10–20 epitope containing regions in this protein. We have recently suggested that a systematic single-residue substitution analysis involving all 20 naturally occurring amino acids followed by statistical ANOVA provides a more sensitive measure of antibody-antigen interaction than a mere signal strength analysis. The large number of peptide fields in the array allowed synthesis of 300 single-substituted variants of *each* of the 595 different linear 15-mer peptides that can be generated from HSA. We used this combined single-residue substitution and ANOVA to generate an automated and highly efficient analysis of this massive amount of data and to pinpoint residues in the original HSA-sequence that are important for binding of polyclonal anti-HSA antibodies. Furthermore, a measure of the relative contribution of each individual position within a given epitope was obtained by calculating the Rq values. The strength of the signals obtained after binding of antibodies to peptide fields in the arrays depends not only on the concentration and affinity of the antibodies but also on the density, purity, and sterical availability of the displayed peptides. The ANOVA only examines distribution of variance, which is independent on the strength of the signals. Thus, weak – but consistent - signals can still be revealed as very specific interactions. This is vividly illustrated by the e.g. the epitope found around 15-mer # 93–100 where the signals are relatively low, around 50 AU (Table S1). However, the HSD analysis clearly identifies amino positions in this region that are significant (p<0.01) for binding of the antibody. The analyses pinpointed the residue positions in the native sequences that are important for binding of the antibodies and it also indicated to which extent specific amino acids can substitute each other in these positions. Inspection of a large number of PSSM’s have revealed a remarkable specificity of each amino acid in the sense that even conservative substitutions in general leads to considerable attenuation of the signals. In some regions of HSA, the residues identified as significant for epitope binding are clustered over longer contiguous sequence stretches, making distinction between different epitopes somewhat ambiguous. By inspection of the way the regions with important amino acids slide through the window of deduced “epitopes” (see rightmost column of Fig. 3a and b) one can get a fairly good idea about where one epitope stops and a new one begins. A statistical measure of the importance of each amino acid was judged by calculating the Rq value. However, a definitive distinction between epitopes in closely positioned clusters of important amino acids cannot be made when using polyclonal antibodies, since the putatively long epitope regions may correspond to paratopes of one or several different antibody clones. Our previous experience with monoclonal antibodies and shorter peptides used for polyclonal antibody binding is that the linear epitopes identified are usually in the range from 4–10 amino acids (data not shown). Based on this, we would suggest that the long clusters of significant residues are caused by two or more epitopes leading to a total of 24 epitope candidates identified with the polyclonal rabbit anti-HSA antibody used.

The vast majority of the more than 200.000 fields in the peptide array were reserved for synthesis of single-residue substituted variants of all possible 15-mer peptides in HSA. The advantage of this approach is that any peptide binding to the antibody can be epitope-mapped at the single-residue level after synthesis of only one peptide array. The entire analysis required only a few micrograms of polyclonal immunoglobulin, and with monoclonal antibodies an array detected by immunofluorescence can in principle be probed consuming antibodies in the nanogram range. In the array shown here, single-residue substitutions were performed on all 15-mer peptides from the target protein and with all 20 common amino acids. This abundance gives highly significant identification of epitope residues, but for most practical purposes a less rigorous regime, e.g. in which every third or fourth 15-mer is analyzed would probably be sufficient. This would allow detailed single-array epitope screenings of protein sequences with a length of several thousand amino acid residues. Substitution analysis with less than 20 different amino acids will give good results in many cases, but as the number of substitutions is decreased, there is an increasing risk that weak epitope residues are overlooked. The optimal balance between number of substitutions and number of fields used in the array depends on the signal to noise ratio in the individual arrays and on the desired resolution of the epitope structures. In cases, where epitope screening of many proteins are needed, it is more rational to use one high-density peptide array for initial screening of the proteins for antibody-binding peptides and then to make a second array in which the epitope regions in these peptides are examined in detail using the single-residue substitution analysis. In this way all e.g. 15-mer peptides from hundreds of HSA-sized proteins can be analyzed in the first array, and in a second array more than a thousand epitope candidates from the first array can be subjected to exhaustive single-residue substitution analysis.

As demonstrated in this report, multiple linear antibody epitopes in a medium sized protein can be detected and characterized using data obtained from a single high-density peptide array. The technique can be useful in identification of disease specific epitopes and of epitopes for paired antibodies to be used in immunoassays. Furthermore, it can be useful in detailed mapping of epitopes for industrial antibodies and in systematic searches for cross-reacting epitopes in relevant proteomes. Although peptide epitopes found in arrays of linear peptide chains are linear by definition, it remains to be demonstrated to what extent the epitopes found in our high-density peptide arrays actually are parts of more complicated conformational epitopes. Simple constraining, e.g. by intra-chain cyclization, of the peptides in the array may increase the chances for mimicking of tertiary structures and thereby for identification of more elements from conformational epitopes [10], [26]. The large number of peptide fields available in the high-density arrays allows for synthesis of myriads of peptides whose sequences are combinations of shorter sequences systematically picked from different regions of a protein antigen, and it may be that further epitopes can be revealed using such peptides with or without structure constraints.

The quality of the peptides synthesized in the arrays will influence the signal intensities recorded after binding of the probes, and with the short coupling cycles used during synthesis of the array it is expected that long peptides and/or sterically hindered peptides will be synthesized with low purity. For peptide ligands with high-affinity binding this may be a minor problem, since even small amounts of correctly synthesized peptide in a field may be adequate for generation of a signal, but for low-affinity ligands and in assays where a high purity of the peptides are required, a low yield of the peptide target may be an impediment. The extremely low amount of peptide per unit surface area in the arrays makes it difficult to make reliable assays of the peptide quality. MALDI-MS analysis of the array peptides is a distinct possibility for achievement of such assays, and we are currently working along these lines with a view to establishment of techniques for validation of micro-array syntheses.

In conclusion, we have presented an application of a high-density peptide microarray technology whose capacity in terms of numbers greatly exceeds the limits of previously published arrays of synthetic peptides with predefined sequences. This technology can reduce the workload and the price for production of large peptide arrays, and we expect it to become a useful high-throughput tool for the identification and mapping of linear peptide epitopes.

## Supporting Information

Figure S1 **Native Protein Sequences.** The sequences of **HSA, RSA** and **BSA**. Underlined residues indicate epitope candidates as judged by signal intensities. Bold indicates residues important for binding as judged by Rq.
(EPS)

Figure S2 **Position Specific Scoring Matrix (PSSM).** An example of a PSSM for single-residue scans of one of the 15-mer peptides from HSA. The n-terminal of the selected 15-mer is located at position 396 in the HSA-sequence. The title row lists the residues in the native peptide. The next 20 rows depict the signals obtained from peptides singly substituted with the residue shown in the title column. The next row shows the mean values calculated from the 20 substitutions. Signals are expressed as percentages of the signal obtained with the native sequence. For each column, mean values deviating significantly from 100 indicate positions that contribute to binding of the antibody. The bottom row shows the native amino acids that have been identified as being significant (p<0.01) for binding by the following HSD post hoc analysis.
(EPS)

Table S1 Summarization of data. Column 1: The position of the peptide’s N-terminal residue in the native protein, Column 2: The peptide sequence, Column 3: The signal intensity after background subtraction, Column 4: The standard deviation obtained from five copies of the peptide, Column 5: The F-value obtained by the ANOVA from each complete peptide scan, Column 6: The η^2^-value obtained by the ANOVA from each complete peptide scan. Boldface values in Column 5 and 6 indicate significant values (p<0.01, η^2^>0.4) values. Column 7: Residues identified by Tukey’s HSD, Column 8–22 Rq-values from the 15 residues in the given peptide.
(PDF)
