# Supplementary material for: Identification and Mapping of Linear Antibody Epitopes in Human Serum Albumin Using High-Density Peptide Arrays
Source: PLoS One. 2013 Jul 23;8(7):e68902. doi: 10.1371/journal.pone.0068902 (PMC3720873; doi:10.1371/journal.pone.0068902)
Supplement: Table S1 — Summarization of data. Column 1: The position of the peptide’s N-terminal residue in the native protein, Column 2: The peptide sequence, Column 3: The signal intensity after background subtraction, Column 4: The standard deviation obtained from five copies of the peptide, Column 5: The F-value obtained by the ANOVA from each complete peptide scan, Column 6: The η2-value obtained by the ANOVA from each complete peptide scan. Boldface values in Column 5 and 6 indicate significant values (p<0.01, η2>0.4) values. Column 7: Residues identified by Tukey’s HSD, Column 8–22 Rq-values from the 15 residues in the given peptide. (PDF) [file pone.0068902.s003.pdf]

15-mer #	Sequence	Signal (AU)	SD	F	η^2	Tukey (HSD)	Rq1	Rq2	Rq3	Rq4	Rq5	Rq6	Rq7	Rq8	Rq9	Rq10	Rq11	Rq12	Rq13	Rq14	Rq15
1	MKWVTFISLLFLFSS	7	31	1.0	0.05	---------------	0.0	0.2	0.0	0.2	0.0	0.1	0.1	0.0	0.2	0.2	0.3	0.2	0.1	0.2	0.2
2	KWVTFISLLFLFSSA	8	31	2.4	0.11	---------------	0.2	0.4	0.0	0.0	0.2	0.3	0.0	0.4	0.5	0.4	0.3	0.0	0.0	0.0	0.4
3	WVTFISLLFLFSSAY	5	6	2.5	0.11	---------------	0.0	0.0	0.0	0.0	0.0	0.2	0.0	0.0	0.0	0.0	0.0	0.0	0.1	0.0	0.0
4	VTFISLLFLFSSAYS	7	8	**3.6**	0.15	---------------	0.0	0.2	0.0	0.0	0.5	0.0	0.0	0.0	0.0	0.0	0.0	0.2	0.0	0.2	0.0
5	TFISLLFLFSSAYSR	4	5	3.2	0.14	---------------	0.0	0.0	0.0	0.2	0.0	0.0	0.0	0.0	0.0	0.0	0.2	0.0	0.0	0.0	0.0
6	FISLLFLFSSAYSRG	5	8	**4.2**	0.17	---------------	0.0	0.0	0.2	0.0	0.0	0.0	0.0	0.0	0.4	0.3	0.0	0.0	0.4	0.0	0.0
7	ISLLFLFSSAYSRGV	6	7	3.3	0.14	---------------	0.1	0.0	0.0	0.0	0.0	0.0	0.0	0.3	0.4	0.4	0.0	0.1	0.0	0.1	0.1
8	SLLFLFSSAYSRGVF	12	6	2.6	0.11	---------------	0.2	0.0	0.0	0.0	0.0	0.0	0.5	0.4	0.1	0.0	0.4	0.0	0.0	0.0	0.4
9	LLFLFSSAYSRGVFR	5	4	2.0	0.09	---------------	0.0	0.0	0.0	0.0	0.0	0.1	0.1	0.0	0.1	0.1	0.0	0.0	0.0	0.1	0.0
10	LFLFSSAYSRGVFRR	10	8	3.0	0.13	---------------	0.0	0.0	0.0	0.0	0.0	0.4	0.0	0.0	0.4	0.1	0.0	0.1	0.5	0.0	0.0
11	FLFSSAYSRGVFRRD	7	5	1.4	0.06	---------------	0.0	0.0	0.0	0.0	0.0	0.0	0.0	0.0	0.0	0.0	0.1	0.3	0.0	0.0	0.0
12	LFSSAYSRGVFRRDA	3	7	**4.5**	0.18	---------------	0.2	0.0	0.0	0.1	0.1	0.0	0.0	0.0	0.0	0.0	0.0	0.0	0.0	0.0	0.1
13	FSSAYSRGVFRRDAH	-7	8	**3.9**	0.16	---------------	0.0	0.0	0.0	0.0	0.2	0.0	0.5	0.1	0.2	0.0	0.4	0.4	0.0	0.1	0.0
14	SSAYSRGVFRRDAHK	-9	8	**4.1**	0.17	---------------	0.0	0.0	0.0	0.0	0.0	0.0	0.0	0.0	0.0	0.0	0.0	0.1	0.0	0.0	0.0
15	SAYSRGVFRRDAHKS	-11	9	**5.9**	0.23	---------------	0.0	0.1	0.0	0.0	0.0	0.2	0.0	0.0	0.0	0.0	0.7	0.0	0.0	0.1	0.0
16	AYSRGVFRRDAHKSE	-11	9	3.5	0.15	---------------	0.0	0.0	0.0	0.0	0.0	0.1	0.0	0.1	0.4	0.0	0.0	0.8	0.1	0.0	0.4
17	YSRGVFRRDAHKSEV	-13	7	1.5	0.07	---------------	0.0	0.0	0.2	0.2	0.0	0.0	0.2	0.3	0.0	0.0	0.5	0.4	0.0	0.2	0.4
18	SRGVFRRDAHKSEVA	-13	9	1.8	0.08	---------------	0.3	0.1	0.2	0.2	0.0	0.0	0.0	0.0	0.0	0.5	0.5	0.1	0.0	0.1	0.4
19	RGVFRRDAHKSEVAH	-10	7	0.9	0.04	---------------	0.0	0.0	0.0	0.0	0.0	0.0	0.0	0.0	0.1	0.0	0.0	0.0	0.0	0.0	0.1
20	GVFRRDAHKSEVAHR	-18	10	0.7	0.03	---------------	0.3	0.3	0.2	0.3	0.2	0.1	0.1	0.1	0.6	0.2	0.1	0.0	0.1	0.3	0.2
21	VFRRDAHKSEVAHRF	-15	8	2.0	0.09	---------------	0.3	0.1	0.4	0.2	0.1	0.4	0.5	0.7	0.2	0.0	0.6	0.4	0.3	0.0	0.0
22	FRRDAHKSEVAHRFK	-20	11	0.9	0.04	---------------	0.7	0.3	0.2	0.5	0.4	0.8	0.5	0.4	0.4	0.3	0.4	0.8	0.4	0.3	0.6
23	RRDAHKSEVAHRFKD	-18	12	2.9	0.13	---------------	0.1	0.1	0.1	0.2	0.1	0.5	0.0	0.0	0.0	0.0	0.2	0.1	0.0	0.9	0.0
24	RDAHKSEVAHRFKDL	-15	12	1.4	0.07	---------------	0.3	0.0	0.1	0.2	0.2	0.2	0.0	0.0	0.0	0.4	0.5	0.0	0.4	0.3	0.0
25	DAHKSEVAHRFKDLG	8	8	**19.2**	**0.49**	---------------	0.4	0.4	0.0	0.0	0.0	0.0	0.2	0.1	0.0	0.5	0.0	0.3	0.0	0.0	0.0
26	AHKSEVAHRFKDLGE	61	9	**65.8**	**0.76**	--------RFKDLGE	0.3	0.0	0.0	0.1	0.6	0.1	0.2	0.0	1.0	0.0	1.4	0.0	0.0	0.0	0.0
27	HKSEVAHRFKDLGEE	94	16	**54.8**	**0.73**	--------FKDLGEE	0.0	0.0	0.0	0.3	0.0	0.1	0.0	0.5	0.0	1.6	0.0	2.9	2.6	2.3	1.4
28	KSEVAHRFKDLGEEN	103	15	**76.1**	**0.79**	-------FKDLGEE-	0.0	0.0	0.2	0.1	0.1	0.0	0.6	0.0	1.3	0.0	3.6	3.2	2.7	1.9	0.1
29	SEVAHRFKDLGEENF	120	21	**65.7**	**0.76**	------FKDLGEE--	0.2	0.4	0.2	0.0	0.0	0.5	3.6	1.4	3.9	3.6	3.1	2.3	1.4	0.0	0.2
30	EVAHRFKDLGEENFK	104	22	**57.5**	**0.74**	-----FKDLGEE---	0.0	0.0	0.0	0.0	0.3	3.2	1.1	3.1	3.1	2.5	2.5	1.8	0.0	0.5	0.0
31	VAHRFKDLGEENFKA	91	15	**67.2**	**0.77**	----FKDLGEE----	0.0	0.3	0.0	0.2	3.3	1.2	3.4	3.2	3.0	2.8	2.1	0.0	0.4	0.0	0.0
32	AHRFKDLGEENFKAL	59	23	**66.5**	**0.77**	---F-DLGEE-----	0.0	0.0	0.0	2.5	0.5	0.0	2.5	2.3	2.4	1.7	0.0	0.0	0.0	0.0	0.0
33	HRFKDLGEENFKALV	73	24	**61.2**	**0.75**	--F-DLGEE------	0.0	0.0	0.0	0.9	0.0	0.0	2.7	2.8	2.2	0.2	0.3	0.0	0.1	0.1	0.0
34	RFKDLGEENFKALVL	77	18	**58.0**	**0.74**	-F-DLGEE-------	0.1	2.8	0.4	0.0	2.9	2.5	2.6	2.2	0.0	0.4	0.0	0.0	0.0	0.0	0.2
35	FKDLGEENFKALVLI	90	21	**40.8**	**0.67**	F-DLGEE--------	2.2	0.2	2.4	2.4	2.3	2.4	2.3	0.8	0.5	0.0	0.4	0.5	0.0	0.4	0.5
36	KDLGEENFKALVLIA	33	8	**9.5**	0.32	---------------	0.0	1.0	0.7	0.8	1.0	1.1	1.0	0.8	0.0	0.9	0.9	0.0	1.0	1.0	0.5
37	DLGEENFKALVLIAF	53	13	**16.2**	**0.44**	DLGEEN--AL--I--	1.2	1.2	1.5	1.8	2.2	1.7	0.5	0.0	1.6	1.4	0.0	0.9	1.0	0.9	0.5
38	LGEENFKALVLIAFA	28	6	**10.1**	0.33	---------------	0.8	0.8	1.1	1.3	0.9	0.0	0.0	0.8	0.6	0.0	0.4	0.3	0.6	0.4	0.4
39	GEENFKALVLIAFAQ	15	6	**4.3**	0.17	---------------	0.4	0.9	1.1	0.6	0.3	0.0	0.2	0.0	0.0	0.0	0.1	0.2	0.0	0.3	0.4
40	EENFKALVLIAFAQY	11	6	**4.7**	0.19	---------------	0.2	0.5	0.4	0.0	0.0	0.3	0.0	0.0	0.0	0.0	0.0	0.0	0.0	0.5	0.0
41	ENFKALVLIAFAQYL	7	6	3.3	0.14	---------------	0.0	0.2	0.2	0.0	0.2	0.0	0.0	0.0	0.0	0.4	0.1	0.1	0.4	0.3	0.2
42	NFKALVLIAFAQYLQ	-2	7	1.8	0.08	---------------	0.0	0.1	0.0	0.0	0.0	0.0	0.0	0.0	0.0	0.1	0.0	0.0	0.0	0.0	0.0
43	FKALVLIAFAQYLQQ	-2	5	1.2	0.06	---------------	0.1	0.0	0.0	0.0	0.0	0.0	0.0	0.1	0.1	0.0	0.0	0.1	0.0	0.1	0.0
44	KALVLIAFAQYLQQC	-6	8	1.2	0.05	---------------	0.0	0.4	0.1	0.1	0.2	0.1	0.1	0.5	0.2	0.0	0.1	0.3	0.0	0.3	0.3
45	ALVLIAFAQYLQQCP	3	6	1.8	0.08	---------------	0.0	0.0	0.1	0.0	0.0	0.0	0.0	0.0	0.0	0.1	0.2	0.1	0.1	0.1	0.0
46	LVLIAFAQYLQQCPF	1	7	1.8	0.08	---------------	0.0	0.0	0.0	0.0	0.0	0.0	0.0	0.0	0.0	0.0	0.0	0.0	0.0	0.0	0.0

47	VLIAFAQYLQQCPFE	4	3	2.5	0.11	----------------	0.0	0.0	0.0	0.0	0.0	0.0	0.0	0.0	0.0	0.0	0.0	0.0	0.0	0.0	0.0
48	LIAFAQYLQQCPFED	13	6	1.0	0.05	----------------	0.0	0.0	0.0	0.4	0.0	0.1	0.0	0.0	0.0	0.0	0.0	0.0	0.1	0.0	0.0
49	IAFAQYLQQCPFEDH	9	5	**4.4**	0.18	----------------	0.2	0.3	0.4	0.0	0.5	0.2	0.5	0.3	0.3	0.0	0.1	0.3	0.3	0.5	0.0
50	AFAQYLQQCPFEDHV	7	7	**5.0**	0.20	----------------	0.2	0.1	0.0	0.1	0.0	0.3	0.1	0.0	0.0	0.0	0.3	0.0	0.1	0.0	0.2
51	FAQYLQQCPFEDHVK	1	8	**5.0**	0.20	----------------	0.0	0.0	0.0	0.0	0.0	0.0	0.0	0.0	0.0	0.0	0.0	0.1	0.0	0.1	0.0
52	AQYLQQCPFEDHVKL	-1	9	**8.5**	0.29	----------------	0.0	0.0	0.0	0.0	0.0	0.0	0.0	0.0	0.0	0.0	0.0	0.0	0.0	0.0	0.0
53	QYLQQCPFEDHVKLV	-1	5	**8.2**	0.29	----------------	0.0	0.0	0.0	0.0	0.0	0.0	0.0	0.0	0.0	0.0	0.0	0.0	0.0	0.0	0.0
54	YLQQCPFEDHVKLVN	4	6	**8.5**	0.30	----------------	0.2	0.2	0.0	0.1	0.1	0.2	0.4	0.0	0.3	0.0	0.0	0.0	0.0	0.2	0.3
55	LQQCPFEDHVKLVNE	1	6	**4.2**	0.17	----------------	0.0	0.0	0.1	0.0	0.0	0.1	0.0	0.1	0.0	0.1	0.0	0.1	0.1	0.0	0.0
56	QQCPFEDHVKLVNEV	6	2	**11.3**	0.36	----------------	0.0	0.0	0.0	0.0	0.3	0.6	0.2	0.0	0.2	0.0	0.5	0.6	0.1	0.0	0.3
57	QCPFEDHVKLVNEVT	108	19	**74.3**	**0.79**	----------LVNEVT	0.0	0.0	0.0	0.3	0.7	0.2	0.0	0.3	0.0	2.9	3.0	3.6	3.7	3.4	1.0
58	CPFEDHVKLVNEVTE	140	23	**80.1**	**0.80**	---------LVNEVTE	0.0	0.0	0.3	0.3	0.0	0.0	0.2	0.0	3.0	3.3	3.8	3.7	3.5	1.2	2.0
59	PFEDHVKLVNEVTEF	508	42	**67.7**	**0.77**	--------LVNEVTEF	0.0	0.1	0.2	0.3	0.0	0.3	0.0	2.5	2.7	3.7	4.4	2.2	2.5	2.0	2.4
60	FEDHVKLVNEVTEFA	518	66	**62.0**	**0.75**	-------LVNEVTEF-	0.2	0.3	0.2	0.1	0.1	0.0	2.4	2.6	3.8	4.4	2.1	2.7	2.1	2.4	0.3
61	EDHVKLVNEVTEFAK	481	61	**60.2**	**0.75**	------LVNEVTEF--	0.2	0.1	0.0	0.2	0.0	2.2	2.6	3.4	4.1	1.9	2.6	2.0	2.4	0.2	0.1
62	DHVKLVNEVTEFAKT	458	40	**65.2**	**0.76**	-----LVNEVTEF---	0.0	0.0	0.2	0.0	2.3	2.5	3.4	4.2	1.6	2.6	2.0	2.4	0.0	0.0	0.1
63	HVKLVNEVTEFAKTC	387	32	**84.7**	**0.81**	----LVNEVTEF----	0.1	0.0	0.0	2.6	2.8	3.8	4.5	1.9	3.0	3.0	2.9	0.0	0.0	0.0	0.0
64	VKLVNEVTEFAKTCV	446	47	**71.4**	**0.78**	---LVNEVTEF-----	0.4	0.0	2.6	2.8	3.6	4.5	2.2	3.2	3.0	3.2	0.2	0.1	0.3	0.3	0.4
65	KLVNEVTEFAKTCVA	368	54	**70.5**	**0.78**	-LVNEVTEF------	0.0	2.6	2.8	3.2	4.1	2.1	2.8	3.2	3.1	0.2	0.0	0.5	0.2	0.1	0.0
66	LVNEVTEFAKTCVAD	452	72	**46.6**	**0.70**	LVNEVTEF--T----	2.2	2.1	2.5	3.2	1.5	2.5	3.6	3.6	0.2	0.5	1.2	0.6	0.9	0.0	0.4
67	VNEVTEFAKTCVADE	217	35	**42.8**	**0.68**	----TEF-KTCV--E	0.3	0.3	0.6	0.2	1.7	3.5	3.7	0.9	1.9	3.4	1.4	2.6	0.2	0.5	1.3
68	NEVTEFAKTCVADES	267	31	**53.6**	**0.72**	----EF-KT-V-DE-	0.0	0.5	0.2	0.6	3.0	4.3	0.7	1.2	3.9	0.7	2.5	0.1	1.1	2.1	0.7
69	EVTEFAKTCVADESA	268	30	**41.3**	**0.67**	---EF-KT-V-DE--	0.2	0.3	0.8	2.8	3.8	0.9	1.3	3.5	0.7	2.2	0.4	1.7	2.3	0.9	0.2
70	VTEFAKTCVADESAE	286	38	**38.6**	**0.65**	--EF-KT-V-DES--	0.3	0.6	3.0	3.9	0.9	1.1	3.4	0.8	2.3	0.5	1.8	3.0	1.2	0.6	0.9
71	TEFAKTCVADESAEN	253	41	**28.9**	**0.59**	-EF--T-V-DES-E-	0.8	2.4	2.9	0.4	0.6	2.6	0.9	1.9	0.5	2.3	3.1	1.7	0.5	1.3	0.3
72	EFAKTCVADESAENC	329	56	**32.7**	**0.62**	-F---CVADESAE-C	0.7	1.2	0.5	0.3	0.7	2.1	1.4	1.2	3.3	3.1	3.2	2.3	3.1	0.6	1.7
73	FAKTCVADESAENCD	309	84	**42.9**	**0.68**	----CVADESAENC-	0.9	0.2	0.3	0.1	2.1	1.1	1.4	2.8	2.8	3.3	3.1	3.7	1.0	2.1	0.8
74	AKTCVADESAENCDK	246	59	**55.9**	**0.73**	---C-ADESAE-CD-	0.0	0.0	0.0	1.7	0.6	1.6	2.8	2.8	3.5	3.2	3.9	1.0	1.8	1.1	0.0
75	KTCVADESAENCDKS	273	61	**55.8**	**0.73**	--C-ADESAE-C---	0.0	0.0	1.7	0.7	1.5	2.9	2.6	3.4	3.2	3.7	0.9	1.9	0.7	0.0	0.2
76	TCVADESAENCDKSL	307	78	**47.0**	**0.70**	-C--DESAE-CD---	0.0	1.7	0.6	0.9	2.6	2.6	3.4	3.2	3.7	0.7	2.2	1.1	0.0	0.8	0.1
77	CVADESAENCDKSLH	190	46	**56.9**	**0.74**	C-ADESAE-CD----	1.6	0.5	1.2	2.4	2.6	3.7	3.3	3.9	0.8	1.9	1.5	0.0	0.4	0.4	0.0
78	VADESAENCDKSLHT	109	21	**56.5**	**0.74**	--DESAE--D-----	0.0	0.9	2.3	2.4	3.2	2.8	3.4	0.3	0.3	1.3	0.0	0.1	0.1	0.0	0.2
79	ADESAENCDKSLHTL	86	12	**54.4**	**0.73**	-DESAE--D------	0.6	2.1	2.4	3.0	2.4	3.1	0.3	0.3	1.3	0.0	0.0	0.1	0.0	0.0	0.0
80	DESAENCDKSLHTLF	350	47	**64.4**	**0.76**	----E--DKSL-TLF	0.6	1.0	0.8	0.8	1.4	0.0	0.0	3.9	2.4	3.2	2.0	0.0	1.7	3.1	2.6
81	ESAENCDKSLHTLFG	311	29	**97.6**	**0.83**	---E--DKSL-TLF-	0.4	0.2	0.2	1.1	0.0	0.1	4.4	2.6	4.1	2.3	0.0	2.1	3.9	3.3	0.2
82	SAENCDKSLHTLFGD	343	37	**94.5**	**0.82**	--E--DKSL-TLF--	0.1	0.1	1.0	0.0	0.0	4.2	2.6	4.1	2.1	0.0	2.0	4.0	3.2	0.1	0.7
83	AENCDKSLHTLFGDK	290	33	**92.0**	**0.82**	----DKSL-TLF-D-	0.0	0.9	0.0	0.0	3.9	2.5	3.8	2.0	0.0	1.9	3.7	3.0	0.1	1.0	0.0
84	ENCDKSLHTLFGDKL	241	26	**79.9**	**0.80**	---DKSL-TLF-D--	0.9	0.1	0.0	3.2	2.3	3.6	1.9	0.0	2.2	3.5	2.9	0.5	1.2	0.0	0.0
85	NCDKSLHTLFGDKLC	159	22	**75.4**	**0.79**	--DKSL-TLF-D---	0.1	0.0	3.1	2.1	3.1	1.8	0.0	2.2	3.1	2.8	0.7	1.6	0.0	0.0	0.1
86	CDKSLHTLFGDKLCT	155	20	**63.2**	**0.76**	-DKSL-TLF-D----	0.0	3.1	2.2	3.0	1.6	0.0	2.1	3.0	2.6	0.6	1.5	0.0	0.0	0.0	0.3
87	DKSLHTLFGDKLCTV	131	23	**60.6**	**0.75**	DKSL-TLF-D-----	2.7	2.2	2.7	1.8	0.0	1.8	2.7	2.5	0.4	1.4	0.0	0.0	0.0	0.2	0.0
88	KSLHTLFGDKLCTVA	-7	4	**4.7**	0.19	----------------	0.0	0.0	0.0	0.4	0.0	0.0	0.0	0.0	0.0	0.5	0.2	0.0	0.0	0.1	0.0
89	SLHTLFGDKLCTVAT	-1	7	**5.0**	0.20	----------------	0.0	0.0	0.0	0.0	0.0	0.0	0.1	0.0	0.0	0.1	0.0	0.0	0.0	0.0	0.0
90	LHTLFGDKLCTVATL	4	5	3.4	0.14	----------------	0.0	0.0	0.5	0.1	0.0	0.3	0.0	0.0	0.3	0.0	0.0	0.0	0.0	0.3	0.4
91	HTLFGDKLCTVATLR	3	7	**7.5**	0.27	----------------	0.0	0.2	0.1	0.3	0.0	0.0	0.0	0.2	0.0	0.0	0.0	0.0	0.0	0.0	0.1
92	TLFGDKLCTVATLRE	19	7	**10.5**	0.34	----------------	0.3	0.3	0.2	0.0	0.9	0.0	0.4	0.0	0.8	1.1	1.2	1.0	0.0	0.9	1.3
93	LFGDKLCTVATLRET	21	6	**15.4**	**0.43**	---------VAT-RE-	0.1	0.1	0.0	0.9	0.0	0.0	0.0	0.6	1.0	1.1	1.0	0.0	1.0	1.5	0.0

94	FGDKLCTVATLRETY	43	16	**23.2**	**0.53**	-------VAT-RE--	0.2	0.0	0.3	0.0	0.0	0.0	0.3	1.4	1.7	1.7	0.0	1.4	1.9	0.1	0.3
95	GDKLCTVATLRETYG	44	10	**26.9**	**0.57**	------VAT-RE---	0.0	0.4	0.0	0.2	0.0	0.4	1.6	1.7	1.9	0.3	1.7	2.0	0.6	0.4	0.0
96	DKLCTVATLRETYGE	66	19	**30.7**	**0.60**	-----VAT-RE----	0.0	0.0	0.3	0.0	0.1	2.0	2.2	2.1	0.9	2.0	2.1	0.8	0.5	0.0	0.7
97	KLCTVATLRETYGEM	56	14	**34.5**	**0.63**	----VATLRET--E-	0.0	0.3	0.0	0.6	2.4	2.2	2.4	1.2	2.1	2.4	1.1	0.7	0.1	1.3	0.0
98	LCTVATLRETYGEMA	56	14	**25.6**	**0.56**	---VATLRET--E--	0.0	0.0	0.6	1.8	1.9	2.0	1.0	1.8	2.0	1.0	0.5	0.2	1.2	0.0	0.0
99	CTVATLRETYGEMAD	36	10	**15.1**	**0.43**	--VATLRET------	0.0	0.4	1.6	1.4	1.4	1.0	1.3	1.5	1.3	0.5	0.0	0.7	0.0	0.0	0.0
100	TVATLRETYGEMADC	50	12	**14.2**	**0.41**	-VATLRET-------	0.4	1.5	1.7	1.7	1.2	1.6	1.6	1.3	0.6	0.1	0.7	0.0	0.0	0.3	0.2
101	VATLRETYGEMADCC	44	19	**4.7**	0.19	---------------	1.2	0.7	1.0	0.9	1.0	1.5	1.2	0.7	0.5	1.3	0.5	0.0	0.9	0.8	1.1
102	ATLRETYGEMADCCA	24	5	**4.0**	0.17	---------------	0.2	0.2	0.7	0.1	0.9	0.4	0.6	1.2	1.4	0.7	0.6	0.8	0.9	0.9	0.3
103	TLRETYGEMADCCAK	20	4	**8.8**	0.30	---------------	0.5	0.5	0.0	1.2	0.5	0.7	0.6	1.5	0.7	0.5	1.2	1.1	1.1	0.1	0.0
104	LRETYGEMADCCAKQ	32	16	**13.5**	**0.40**	------E--DCC--Q	0.4	0.0	0.8	0.0	0.0	0.5	1.7	0.3	0.2	1.4	1.1	1.3	0.5	0.0	1.1
105	RETYGEMADCCAKQE	49	14	**14.6**	**0.42**	-----E--DCC--QE	0.1	0.2	0.0	0.4	0.7	1.5	0.7	0.7	1.8	1.9	2.0	0.9	0.2	1.5	1.3
106	ETYGEMADCCAKQEP	88	36	**20.4**	**0.50**	----E--DCCA-QE-	0.0	0.0	0.3	0.0	1.2	0.3	0.4	1.6	2.1	2.1	1.3	0.9	2.0	1.6	0.5
107	TYGEMADCCAKQEPE	125	56	**18.5**	**0.48**	---E--DCCAKQE-E	0.5	0.5	0.8	1.6	0.6	0.7	1.7	2.6	2.6	1.3	1.0	2.5	2.2	0.9	1.4
108	YGEMADCCAKQEPER	108	36	**14.7**	**0.42**	--E--DCCAKQE-E-	0.4	0.5	1.2	0.4	0.9	1.7	2.1	2.3	1.2	1.2	2.3	1.6	0.5	1.4	0.1
109	GEMADCCAKQEPERN	141	44	**14.3**	**0.41**	-E--DCCAKQEPE--	0.7	1.3	0.6	0.5	1.6	2.2	2.3	1.7	1.7	2.6	1.9	1.2	2.1	0.5	0.6
110	EMADCCAKQEPERNE	155	58	**19.7**	**0.49**	EM-DCCA-QE-E---	1.5	1.2	0.5	1.8	2.6	2.7	1.3	0.9	2.3	1.5	0.8	1.5	0.2	0.0	0.5
111	MADCCAKQEPERNEC	110	39	**21.2**	**0.51**	---C-AKQ-PE----	0.0	0.0	1.0	1.1	0.9	1.3	1.6	2.3	0.9	1.2	1.9	0.2	0.0	1.0	0.3
112	ADCCAKQEPERNECF	167	71	**12.5**	0.38	---------------	0.3	1.1	1.7	1.0	1.7	2.0	2.6	1.4	1.7	2.4	0.7	0.8	1.1	1.3	0.5
113	DCCAKQEPERNECFL	127	55	**17.7**	**0.47**	-C--KQEPE--E---	0.5	1.2	0.6	1.0	1.5	2.5	1.1	1.7	2.2	0.1	0.0	1.3	0.9	0.3	0.2
114	CCAKQEPERNECFLQ	112	48	**15.6**	**0.43**	C--KQEPE--E----	1.0	0.2	0.9	1.2	2.1	1.2	1.9	2.1	0.0	0.3	1.7	0.9	0.5	0.5	0.0
115	CAKQEPERNECFLQH	36	13	**14.4**	**0.41**	---Q-PE--E-----	0.7	0.6	0.5	1.4	1.0	1.4	1.6	0.2	0.0	1.5	0.4	0.5	0.4	0.1	0.0
116	AKQEPERNECFLQHK	18	8	**21.1**	**0.51**	--Q-PE--E------	0.7	0.2	0.0	1.0	1.1	0.0	0.2	0.0	0.0	0.0	0.3	0.4	0.0	0.0	0.0
117	KQEPERNECFLQHKD	1	3	**12.4**	0.38	---------------	0.0	0.0	0.0	0.1	0.0	0.0	0.0	0.0	0.0	0.0	0.0	0.0	0.0	0.0	0.0
118	QEPERNECFLQHKDD	3	4	**6.5**	0.24	---------------	0.0	0.0	0.0	0.0	0.1	0.0	0.3	0.0	0.0	0.1	0.1	0.0	0.0	0.1	0.0
119	EPERNECFLQHKDDN	3	2	**6.4**	0.24	---------------	0.0	0.0	0.2	0.0	0.0	0.4	0.1	0.3	0.0	0.1	0.0	0.0	0.0	0.0	0.0
120	PERNECFLQHKDDNP	1	4	**11.4**	0.36	---------------	0.0	0.0	0.0	0.0	0.0	0.0	0.0	0.0	0.0	0.0	0.0	0.2	0.1	0.0	0.0
121	ERNECFLQHKDDNPN	4	4	**11.7**	0.37	---------------	0.0	0.0	0.0	0.0	0.0	0.5	0.3	0.0	0.0	0.0	0.6	0.3	0.4	0.1	0.0
122	RNECFLQHKDDNPNL	23	15	**23.0**	**0.53**	---------D-NPNL	0.0	0.0	0.5	0.0	0.0	0.2	0.0	0.0	0.1	1.3	0.8	1.3	1.3	1.3	1.3
123	NECFLQHKDDNPNLP	79	48	**17.6**	**0.46**	--------DDNPNLP	0.2	0.6	0.0	0.4	0.8	0.7	0.0	0.5	1.6	1.0	1.9	1.9	1.9	1.9	1.4
124	ECFLQHKDDNPNLPR	181	103	**14.5**	**0.42**	--------DNPNLPR	0.6	0.0	0.1	0.2	0.0	0.0	0.0	0.6	1.0	1.4	1.6	1.8	1.2	1.3	1.0
125	CFLQHKDDNPNLPRL	248	133	**18.1**	**0.47**	------DDNPNLP--	0.0	0.2	0.4	0.3	0.0	0.0	1.0	1.3	1.2	1.6	2.0	1.5	1.6	0.8	0.4
126	FLQHKDDNPNLPRLV	301	153	**11.5**	0.36	---------------	0.4	0.0	0.5	0.0	0.0	0.9	0.8	1.0	1.4	1.8	1.4	1.5	0.8	0.4	0.3
127	LQHKDDNPNLPRLVR	282	149	**17.0**	**0.45**	----DDNPNLP----	0.2	0.0	0.0	0.0	1.0	1.1	1.3	1.4	2.0	1.7	1.9	0.9	0.4	0.6	0.0
128	QHKDDNPNLPRLVRP	259	137	**12.8**	0.39	---------------	0.0	0.0	0.0	0.5	0.6	1.1	0.8	1.4	1.2	1.2	0.7	0.0	0.0	0.0	0.2
129	HKDDNPNLPRLVRPE	199	95	**11.3**	0.36	---------------	0.0	0.0	0.1	0.2	0.6	0.6	1.2	1.0	1.0	0.2	0.3	0.0	0.0	0.4	0.0
130	KDDNPNLPRLVRPEV	237	94	**12.5**	0.38	---------------	0.0	0.4	0.5	1.0	0.8	1.4	1.5	1.3	0.7	0.5	0.2	0.0	0.2	0.0	0.0
131	DDNPNLPRLVRPEVD	304	107	**8.6**	0.30	---------------	0.3	0.7	1.1	0.9	1.5	1.5	1.6	1.2	0.7	0.5	0.2	0.7	0.0	0.1	0.0
132	DNPNLPRLVRPEVDV	222	94	**5.5**	0.21	---------------	1.0	1.3	1.3	1.8	1.8	2.1	1.6	1.6	1.5	0.9	1.4	1.2	0.9	0.8	0.5
133	NPNLPRLVRPEVDVM	159	78	**9.8**	0.32	---------------	0.6	0.4	0.8	1.5	1.5	1.3	1.2	1.3	0.5	1.2	1.0	0.0	0.3	0.0	0.0
134	PNLPRLVRPEVDVMC	109	47	**9.7**	0.32	---------------	0.7	0.6	1.3	1.4	1.6	1.9	1.7	0.9	1.8	1.8	0.8	1.1	0.5	0.3	0.3
135	NLPRLVRPEVDVMCT	79	30	**17.5**	**0.46**	--PRLVRPE-D----	0.0	1.0	1.3	1.4	1.8	1.9	1.3	1.9	1.6	0.8	1.1	0.0	0.0	0.0	0.2
136	LPRLVRPEVDVMCTA	61	23	**14.6**	**0.42**	-PRLVRPEVD-----	0.9	1.1	1.2	1.8	1.7	1.2	1.9	2.0	1.1	1.2	0.4	0.0	0.0	0.5	0.0
137	PRLVRPEVDVMCTAF	50	19	**17.2**	**0.46**	--LVRPEVD------	0.4	0.5	1.3	1.3	1.4	1.5	2.6	1.6	2.1	0.8	0.6	0.0	0.8	0.0	0.7
138	RLVRPEVDVMCTAFH	23	8	**30.5**	**0.60**	-----EVD-------	0.0	0.7	0.7	0.9	1.0	0.0	1.6	2.1	0.4	0.6	0.0	0.9	0.2	0.3	0.0
139	LVRPEVDVMCTAFHD	35	9	**15.3**	**0.43**	----EVD---T----	0.5	0.5	0.3	0.6	1.5	1.1	1.4	0.7	0.7	0.2	1.0	0.2	0.8	0.0	0.6
140	VRPEVDVMCTAFHDN	17	3	**9.6**	0.32	---------------	0.0	0.0	0.0	0.7	0.2	0.6	0.3	0.2	0.0	0.5	0.1	0.4	0.0	0.3	0.0

141	RPEVDVMCTAFHDNE	24	5	**9.8**	0.33	----------------	0.0	0.0	0.7	0.3	0.7	0.4	0.4	0.1	0.7	0.0	0.4	0.0	0.3	0.0	0.0
142	PEVDVMCTAFHDNEE	16	3	**7.8**	0.28	----------------	0.0	0.5	0.3	0.5	0.3	0.3	0.2	0.5	0.0	0.5	0.0	0.3	0.1	0.0	0.0
143	EVDVMCTAFHDNEET	36	7	**12.2**	0.37	----------------	0.4	0.2	0.6	0.1	0.2	0.2	0.6	0.4	0.9	0.0	0.8	0.8	0.6	0.6	0.2
144	VDVMCTAFHDNEETF	156	36	**40.0**	**0.66**	-------F-DNEETF	0.0	0.5	0.0	0.0	0.3	0.7	0.8	2.0	0.0	2.5	2.5	2.3	2.4	1.5	1.9
145	DVMCTAFHDNEETFL	237	57	**49.7**	**0.71**	------F-DNEETF-	0.0	0.0	0.0	0.0	0.1	0.3	1.5	0.0	2.6	2.6	2.2	2.4	1.3	1.8	0.4
146	VMCTAFHDNEETFLK	336	78	**45.1**	**0.69**	-----F-DNEETF--	0.0	0.0	0.0	0.5	0.3	1.4	0.0	2.9	2.9	2.5	2.8	1.7	1.9	0.7	0.3
147	MCTAFHDNEETFLKK	415	82	**55.0**	**0.73**	----F-DNEETF---	0.1	0.3	0.2	0.6	1.1	0.0	3.3	3.3	2.8	3.2	2.0	2.1	0.7	0.0	0.2
148	CTAFHDNEETFLKKY	486	78	**64.1**	**0.76**	---F-DNEETF----	0.0	0.4	0.6	1.4	0.0	3.8	3.8	3.2	3.7	2.3	2.5	0.9	0.2	0.4	0.4
149	TAFHDNEETFLKKYL	528	86	**67.7**	**0.77**	--F-DNEETFL----	0.0	0.4	1.4	0.0	3.8	3.8	3.0	3.7	2.3	2.7	1.2	0.2	0.3	0.4	0.4
150	AFHDNEETFLKKYLY	420	102	**51.5**	**0.72**	-F-DNEETFL-----	0.1	1.1	0.0	3.0	3.0	2.6	3.0	2.1	2.1	1.0	0.0	0.0	0.6	0.0	0.2
151	FHDNEETFLKKYLYE	291	81	**40.7**	**0.67**	--DNEETF-------	0.6	0.0	2.5	2.5	2.1	2.4	1.8	1.7	0.6	0.0	0.0	0.3	0.2	0.1	0.0
152	HDNEETFLKKYLYEI	102	46	**20.5**	**0.50**	-DNEETFL-------	0.1	1.8	1.8	1.7	1.8	1.7	1.5	1.3	0.0	0.3	0.3	0.2	0.3	0.2	0.0
153	DNEETFLKKYLYEIA	134	62	**27.8**	**0.58**	DNEETFL--------	2.2	2.2	2.1	2.3	2.1	2.1	1.8	0.4	0.4	0.6	0.5	0.4	0.2	0.2	0.0
154	NEETFLKKYLYEIAR	23	3	**11.5**	0.36	----------------	0.5	1.0	0.9	0.2	0.8	0.5	0.0	0.0	0.4	0.6	0.5	0.7	0.2	0.0	0.0
155	EETFLKKYLYEIARR	10	3	**11.0**	0.35	----------------	0.3	0.4	0.2	0.5	0.4	0.0	0.0	0.3	0.6	0.3	0.8	0.1	0.0	0.0	0.0
156	ETFLKKYLYEIARRH	-2	3	**9.1**	0.31	----------------	0.0	0.0	0.0	0.0	0.0	0.0	0.0	0.0	0.0	0.0	0.0	0.0	0.0	0.0	0.0
157	TFLKKYLYEIARRHP	-6	3	**5.8**	0.22	----------------	0.0	0.0	0.0	0.8	1.0	0.0	0.1	0.0	0.0	0.0	0.1	0.3	0.6	0.6	0.1
158	FLKKYLYEIARRHPY	-6	2	**9.0**	0.31	----------------	0.0	0.0	0.8	0.9	0.0	0.0	0.0	0.0	0.0	0.0	0.3	0.4	1.0	0.0	0.0
159	LKKYLYEIARRHPYF	-8	3	**5.0**	0.20	----------------	0.0	0.7	0.6	0.0	0.0	0.0	0.0	0.0	0.1	0.2	0.3	0.0	0.3	0.0	0.0
160	KKYLYEIARRHPYFY	-8	4	2.5	0.11	----------------	0.6	0.4	0.0	0.0	0.0	0.0	0.0	0.0	0.1	0.0	0.4	0.1	0.0	0.0	0.0
161	KYLYEIARRHPYFYA	-3	5	**7.0**	0.26	----------------	0.0	0.0	0.0	0.0	0.0	0.0	0.0	0.4	0.4	0.0	0.1	0.0	0.0	0.0	0.0
162	YLYEIARRHPYFYAP	2	5	**8.7**	0.30	----------------	0.1	0.1	0.2	0.0	0.0	0.0	0.0	0.0	0.0	0.1	0.2	0.0	0.0	0.0	0.0
163	LYEIARRHPYFYAPE	5	6	**6.3**	0.24	----------------	0.2	0.1	0.3	0.0	0.0	0.0	0.0	0.0	0.0	0.1	0.4	0.1	0.0	0.0	0.3
164	YEIARRHPYFYAPEL	4	3	**6.2**	0.23	----------------	0.2	0.3	0.0	0.0	0.0	0.0	0.0	0.0	0.4	0.2	0.0	0.0	0.0	0.0	0.1
165	EIARRHPYFYAPELL	2	11	**5.1**	0.20	----------------	0.1	0.0	0.0	0.0	0.0	0.0	0.0	0.0	0.0	0.0	0.0	0.0	0.0	0.0	0.0
166	IARRHPYFYAPELLF	6	4	**5.5**	0.21	----------------	0.1	0.0	0.0	0.0	0.0	0.0	0.0	0.0	0.0	0.0	0.0	0.0	0.0	0.2	0.1
167	ARRHPYFYAPELLFF	8	5	**8.2**	0.29	----------------	0.0	0.0	0.0	0.0	0.0	0.3	0.0	0.0	0.0	0.0	0.7	0.0	0.2	0.4	0.5
168	RRHPYFYAPELLFFA	9	4	**10.3**	0.34	----------------	0.0	0.0	0.0	0.0	0.0	0.8	0.0	0.2	0.0	0.0	0.4	0.3	0.8	0.9	0.0
169	RHPYFYAPELLFFAK	9	4	**13.8**	**0.41**	----------------	0.0	0.0	0.2	0.1	0.1	0.1	0.0	0.0	0.0	0.4	0.4	0.9	0.6	0.0	0.0
170	HPYFYAPELLFFAKR	90	30	**21.3**	**0.51**	------PE-LFF-KR	0.3	0.5	0.0	0.2	0.5	0.4	1.9	2.4	0.7	1.1	2.4	2.0	0.7	1.7	2.0
171	PYFYAPELLFFAKRY	96	36	**28.7**	**0.59**	-----PE-LFFAKR-	0.3	0.0	0.2	0.4	0.5	2.2	2.7	0.9	1.2	2.6	2.0	1.1	1.9	2.0	0.0
172	YFYAPELLFFAKRYK	46	18	**26.5**	**0.57**	----PE-LFF-KR--	0.0	0.0	0.0	0.2	1.8	2.0	0.5	1.1	2.0	1.5	0.9	1.6	1.3	0.0	0.0
173	FYAPELLFFAKRYKA	38	13	**17.5**	**0.47**	---PE--FF-K----	0.2	0.1	0.0	1.4	1.6	0.4	0.9	1.7	1.3	0.6	1.3	1.0	0.0	0.0	0.0
174	YAPELLFFAKRYKAA	26	12	**15.0**	**0.43**	--PE-LFF-KR----	0.2	0.2	1.4	0.0	0.3	1.2	0.0	1.1	0.7	1.4	1.2	0.1	0.0	0.1	0.3
175	APELLFFAKRYKAAF	21	11	**15.9**	**0.44**	-PE-LFF-K------	0.0	0.0	0.0	0.5	1.3	0.0	1.0	0.6	1.1	0.7	0.0	0.0	0.0	0.2	0.0
176	PELLFFAKRYKAAFT	71	19	**24.1**	**0.54**	PE-LFF-K-------	2.2	2.0	0.6	2.0	2.1	1.1	1.0	2.1	0.8	0.0	0.1	0.2	0.2	0.0	0.4
177	ELLFFAKRYKAAFTE	5	4	**6.5**	0.24	----------------	0.0	0.4	0.5	0.0	0.5	0.1	0.0	0.0	0.2	0.0	0.2	0.4	0.5	0.2	0.5
178	LLFFAKRYKAAFTEC	1	5	**8.2**	0.29	----------------	0.0	0.1	0.0	0.0	0.0	0.0	0.0	0.0	0.0	0.1	0.1	0.0	0.0	0.0	0.0
179	LFFAKRYKAAFTECC	-2	4	2.7	0.12	----------------	0.0	0.0	0.0	0.1	0.0	0.0	0.0	0.0	0.0	0.0	0.0	0.0	0.0	0.1	0.0
180	FFAKRYKAAFTECCQ	-2	4	**4.1**	0.17	----------------	0.0	0.0	0.0	0.0	0.0	0.1	0.0	0.0	0.1	0.0	0.0	0.0	0.0	0.0	0.0
181	FAKRYKAAFTECCQA	-5	5	**7.9**	0.28	----------------	0.0	0.1	0.0	0.6	0.0	0.0	0.0	0.0	0.0	0.0	0.0	0.1	0.0	0.0	0.0
182	AKRYKAAFTECCQAA	-9	5	**5.7**	0.22	----------------	0.4	0.8	0.6	0.0	0.8	0.0	0.0	0.0	0.0	0.0	0.2	0.2	0.0	0.4	0.0
183	KRYKAAFTECCQAAD	-7	4	**4.8**	0.19	----------------	0.8	0.4	0.0	0.7	0.1	0.2	0.1	0.0	0.0	0.0	0.2	0.0	0.0	0.1	0.0
184	RYKAAFTECCQAADK	-7	6	**5.9**	0.23	----------------	0.8	0.2	0.0	0.0	0.1	0.0	0.3	0.0	0.2	0.4	0.0	0.3	0.1	0.0	0.8
185	YKAAFTECCQAADKA	-3	3	**7.0**	0.26	----------------	0.0	0.0	0.3	0.2	0.0	0.1	0.0	0.0	0.0	0.1	0.0	0.1	0.0	0.0	0.1
186	KAAFTECCQAADKAA	-4	4	**5.2**	0.20	----------------	0.0	0.0	0.1	0.0	0.0	0.0	0.0	0.0	0.0	0.1	0.0	0.0	0.0	0.3	0.0
187	AAFTECCQAADKAAC	9	3	**7.5**	0.27	----------------	0.1	0.4	0.6	0.0	0.0	0.3	0.0	0.5	0.3	0.3	1.0	0.0	0.0	0.3	0.6

188	AFTECCQAADKAACL	8	3	**7.0**	0.26	----------------	0.0	0.1	0.0	0.9	0.2	0.0	0.3	0.2	0.0	1.0	0.0	0.0	0.1	0.3	0.2
189	FTECCQAADKAACLL	10	4	**5.0**	0.20	----------------	0.1	0.3	1.0	0.0	0.0	0.4	0.5	0.2	0.7	0.0	0.0	0.2	0.4	0.2	0.0
190	TECCQAADKAACLLP	7	3	**9.1**	0.31	----------------	0.1	0.0	0.4	0.0	0.2	0.3	0.2	0.0	0.0	0.0	0.0	0.6	0.0	0.0	0.0
191	ECCQAADKAACLLPK	-4	3	**5.8**	0.22	----------------	0.0	0.3	0.0	0.0	0.0	0.1	0.0	0.0	0.1	0.4	0.0	0.1	0.2	0.0	0.0
192	CCQAADKAACLLPKL	-10	6	**5.4**	0.21	----------------	0.3	0.5	0.2	0.4	0.2	0.0	1.3	0.6	0.6	0.6	0.3	0.2	0.8	1.5	0.2
193	CQAADKAACLLPKLD	-2	2	**11.3**	0.36	----------------	0.0	0.0	0.0	0.0	0.0	0.0	0.0	0.0	0.0	0.0	0.0	0.0	0.0	0.0	0.0
194	QAADKAACLLPKLDE	8	5	**19.4**	**0.49**	----------------	0.0	0.0	0.0	0.0	0.0	0.5	0.6	0.0	0.0	0.0	0.4	0.0	0.0	0.0	0.0
195	AADKAACLLPKLDEL	31	15	**27.9**	**0.58**	--D----LLP-LDE-	0.0	0.0	1.1	0.0	0.5	0.9	0.0	1.1	1.3	1.2	0.0	1.5	0.0	0.0	0.8
196	ADKAACLLPKLDELR	30	11	**22.4**	**0.53**	----------LDE--	0.0	0.8	0.0	0.4	0.5	0.0	0.8	0.8	0.8	0.0	1.3	1.4	0.0	0.8	0.0
197	DKAACLLPKLDELRD	35	9	**25.4**	**0.56**	------LP-LDEL--	0.9	0.0	0.5	0.6	0.0	0.9	1.1	1.0	0.0	1.7	1.9	0.0	1.0	0.0	0.6
198	KAACLLPKLDELRDE	23	6	**19.2**	**0.49**	--------LDE----	0.0	0.0	0.1	0.0	0.6	0.7	0.6	0.5	1.2	0.0	0.0	1.0	0.0	0.3	0.5
199	AACLLPKLDELRDEG	32	5	**16.9**	**0.45**	------KLDE-----	0.0	0.1	0.0	0.4	0.5	0.6	1.0	1.6	1.8	1.8	1.0	0.0	0.0	0.3	0.0
200	ACLLPKLDELRDEGK	30	6	**17.3**	**0.46**	-----KLDEL--E--	0.0	0.0	0.4	0.5	0.3	1.1	1.4	1.8	2.1	1.0	0.0	0.5	1.3	0.0	0.0
201	CLLPKLDELRDEGKA	24	5	**15.6**	**0.44**	-----LDE---E---	0.0	0.6	0.4	0.1	0.8	1.4	1.6	1.9	1.0	0.0	0.7	1.3	0.0	0.0	0.0
202	LLPKLDELRDEGKAS	22	3	**7.2**	0.26	----------------	0.4	0.5	0.4	0.6	0.9	1.1	1.3	0.7	0.0	0.8	0.9	0.0	0.0	0.1	0.2
203	LPKLDELRDEGKASS	20	5	**11.1**	0.36	----------------	0.6	0.0	0.6	1.2	1.3	1.5	1.2	0.3	0.3	0.8	0.0	0.0	0.0	0.0	0.0
204	PKLDELRDEGKASSA	7	4	**5.0**	0.20	----------------	0.1	0.0	0.3	0.7	0.8	0.5	0.0	0.4	0.0	0.0	0.0	0.1	0.0	0.0	0.1
205	KLDELRDEGKASSAK	-3	5	**6.9**	0.25	----------------	0.0	0.0	0.0	0.0	0.1	0.0	0.0	0.0	0.2	0.0	0.0	0.1	0.0	0.2	0.0
206	LDELRDEGKASSAKQ	15	4	**11.3**	0.36	----------------	0.1	0.0	0.5	0.1	0.0	0.6	0.0	0.1	0.6	1.0	0.2	0.9	0.7	0.0	0.8
207	DELRDEGKASSAKQR	2	4	**28.1**	**0.58**	----------------	0.2	0.1	0.0	0.0	0.0	0.0	0.0	0.0	0.0	0.2	0.0	0.0	0.0	0.0	0.0
208	ELRDEGKASSAKQRL	2	4	**22.2**	**0.52**	----------------	0.2	0.0	0.0	0.0	0.0	0.0	0.0	0.0	0.0	0.0	0.0	0.0	0.0	0.0	0.0
209	LRDEGKASSAKQRLK	-1	3	**9.0**	0.31	----------------	0.0	0.0	0.0	0.0	0.0	0.0	0.0	0.0	0.0	0.0	0.0	0.0	0.0	0.0	0.0
210	RDEGKASSAKQRLKC	-7	3	**4.1**	0.17	----------------	0.1	0.0	0.0	0.0	0.0	0.0	0.2	0.0	0.0	0.3	0.0	0.4	0.0	0.2	0.5
211	DEGKASSAKQRLKCA	-7	3	**9.1**	0.31	----------------	0.1	0.0	0.1	0.0	0.0	0.0	0.0	0.0	0.8	0.0	0.3	0.0	0.8	0.6	0.0
212	EGKASSAKQRLKCAS	-9	4	3.5	0.15	----------------	0.0	0.0	0.0	0.1	0.0	0.0	0.0	0.6	0.0	0.8	0.1	0.4	0.6	0.0	0.0
213	GKASSAKQRLKCASL	-7	13	1.3	0.06	----------------	0.0	0.0	0.0	0.0	0.0	0.0	0.1	0.0	0.0	0.0	0.0	0.0	0.0	0.0	0.0
214	KASSAKQRLKCASLQ	-9	10	1.1	0.05	----------------	0.3	0.0	0.0	0.0	0.2	0.2	0.0	0.0	0.0	0.0	0.0	0.0	0.1	0.0	0.0
215	ASSAKQRLKCASLQK	-11	8	1.4	0.06	----------------	0.1	0.0	0.0	0.0	0.2	0.3	0.2	0.2	0.5	0.5	0.4	0.3	0.1	0.1	0.3
216	SSAKQRLKCASLQKF	-9	11	1.8	0.08	----------------	0.2	0.0	0.0	0.0	0.0	0.0	0.2	0.3	0.5	0.4	0.0	0.0	0.1	0.1	0.1
217	SAKQRLKCASLQKFG	-11	12	1.4	0.07	----------------	0.1	0.2	0.4	0.0	0.0	0.3	0.3	0.0	0.1	0.3	0.2	0.1	0.5	0.1	0.1
218	AKQRLKCASLQKFGE	-2	12	1.0	0.04	----------------	0.0	0.0	0.0	0.0	0.0	0.0	0.0	0.0	0.0	0.0	0.0	0.0	0.0	0.0	0.0
219	KQRLKCASLQKFGER	-9	10	1.1	0.05	----------------	0.2	0.2	0.2	0.0	0.1	0.0	0.0	0.1	0.0	0.2	0.1	0.1	0.0	0.0	0.0
220	QRLKCASLQKFGERA	1	11	3.5	0.15	----------------	0.0	0.0	0.0	0.0	0.0	0.0	0.0	0.0	0.0	0.0	0.0	0.0	0.0	0.0	0.0
221	RLKCASLQKFGERAF	25	18	**22.9**	**0.53**	---------FGE---	0.0	0.0	0.0	0.0	0.0	0.0	0.0	0.0	0.7	0.0	0.0	0.0	1.0	0.0	0.9
222	LKCASLQKFGERAFK	28	14	**34.5**	**0.63**	-------KFGER-F-	0.0	0.0	0.0	0.0	0.5	0.0	0.0	1.2	0.0	0.0	0.0	1.1	0.0	0.0	0.0
223	KCASLQKFGERAFKA	20	14	**23.4**	**0.54**	------KFGER-F--	0.0	0.0	0.0	0.0	0.0	0.0	1.2	0.0	0.0	0.0	1.1	0.0	1.2	0.0	0.0
224	CASLQKFGERAFKAW	38	13	**19.6**	**0.49**	-----KFGER-F---	0.0	0.3	0.5	0.3	0.3	1.1	2.2	1.5	2.1	1.2	0.1	1.6	0.0	0.2	0.1
225	ASLQKFGERAFKAWA	36	13	**16.6**	**0.45**	----KFGER-F----	0.1	0.2	0.1	0.2	1.2	1.8	1.4	1.6	1.0	0.2	1.2	0.0	0.0	0.2	0.0
226	SLQKFGERAFKAWAV	29	7	**14.9**	**0.42**	----FGER-F-----	0.3	0.2	0.2	0.8	1.4	1.1	1.4	1.1	0.0	1.4	0.0	0.0	0.3	0.0	0.1
227	LQKFGERAFKAWAVA	23	13	**8.7**	0.30	----------------	0.2	0.1	0.2	1.0	1.0	1.0	0.5	0.0	0.9	0.0	0.0	0.0	0.0	0.0	0.0
228	QKFGERAFKAWAVAR	8	11	**10.2**	0.34	----------------	0.0	0.0	0.0	0.0	0.0	0.1	0.0	0.0	0.0	0.0	0.0	0.0	0.0	0.0	0.0
229	KFGERAFKAWAVARL	18	8	**12.4**	0.38	----------------	0.7	1.1	0.9	0.0	0.5	0.0	0.9	0.0	0.0	0.0	0.0	0.0	0.0	0.0	0.0
230	FGERAFKAWAVARLS	12	9	2.5	0.11	----------------	0.3	0.2	0.4	0.0	0.0	0.3	0.0	0.0	0.5	0.0	0.0	0.0	0.0	0.1	0.0
231	GERAFKAWAVARLSQ	1	16	2.2	0.10	----------------	0.0	0.0	0.0	0.0	0.0	0.0	0.0	0.0	0.0	0.0	0.0	0.0	0.0	0.0	0.0
232	ERAFKAWAVARLSQR	-1	15	2.6	0.11	----------------	0.0	0.0	0.0	0.0	0.0	0.0	0.0	0.0	0.1	0.0	0.0	0.0	0.0	0.0	0.0
233	RAFKAWAVARLSQRF	3	10	2.3	0.10	----------------	0.0	0.0	0.0	0.0	0.1	0.0	0.0	0.0	0.0	0.1	0.1	0.2	0.0	0.0	0.0
234	AFKAWAVARLSQRFP	1	13	2.0	0.09	----------------	0.0	0.0	0.0	0.0	0.0	0.1	0.0	0.0	0.0	0.0	0.0	0.0	0.0	0.0	0.0

235	FKAWAVARLSQRFPK	3	10	1.0	0.05	----------------	0.0	0.0	0.1	0.0	0.0	0.0	0.0	0.0	0.0	0.0	0.0	0.2	0.0	0.0	0.2
236	KAWAVARLSQRFPKA	29	22	**12.4**	0.38	----------------	0.0	0.0	0.1	0.2	0.3	0.0	0.1	0.0	0.5	0.0	1.2	0.0	0.0	0.4	0.0
237	AWAVARLSQRFPKAE	21	12	**3.8**	0.16	----------------	0.0	0.1	0.1	0.3	0.3	0.0	0.3	0.3	0.8	0.3	0.8	0.4	0.0	0.7	1.0
238	WAVARLSQRFPKAEF	38	17	**15.8**	**0.44**	---------FP-AE-	0.0	0.0	0.0	0.0	0.0	0.0	0.0	0.8	0.7	1.8	1.3	0.0	1.1	1.7	0.8
239	AVARLSQRFPKAEFA	82	21	**33.5**	**0.62**	--------FPKAEF-	0.0	0.0	0.0	0.0	0.0	0.0	0.5	0.7	1.5	2.3	1.4	2.1	2.7	1.9	0.6
240	VARLSQRFPKAEFAE	124	22	**37.7**	**0.65**	-----QRFPKAEFAE	0.8	0.5	0.2	0.7	0.9	1.3	1.3	1.8	3.3	2.4	3.3	3.9	2.8	1.8	2.0
241	ARLSQRFPKAEFAEV	129	21	**39.3**	**0.66**	----QRFPKAEF-E-	0.0	0.1	0.2	0.4	1.0	1.1	1.5	2.7	1.4	3.0	3.6	2.7	0.9	1.7	0.0
242	RLSQRFPKAEFAEVS	134	23	**44.3**	**0.69**	-----FPKAEFAE--	0.0	0.2	0.0	0.6	0.8	1.3	2.5	1.2	3.1	3.5	2.9	1.2	1.6	0.4	0.1
243	LSQRFPKAEFAEVSK	138	31	**32.0**	**0.61**	----FPKAEFAE---	0.3	0.5	0.7	0.6	1.3	2.4	1.6	2.8	3.4	2.9	1.3	2.1	0.5	0.7	0.2
244	SQRFPKAEFAEVSKL	117	36	**43.7**	**0.68**	----PKAEFAE----	0.0	0.3	0.4	0.9	2.4	1.5	2.7	3.5	2.7	1.3	2.1	0.2	0.2	0.0	0.3
245	QRFPKAEFAEVSKLV	111	22	**38.0**	**0.65**	---PKAEFAE-----	0.6	0.3	0.8	2.2	1.3	3.0	3.3	2.7	1.3	2.1	0.3	0.5	0.0	0.0	0.1
246	RFPKAEFAEVSKLVT	86	18	**39.0**	**0.66**	--PKAEFAE------	0.0	1.0	1.9	1.4	2.4	3.1	2.9	1.5	2.4	0.8	0.6	0.0	0.0	0.2	0.5
247	FPKAEFAEVSKLVTD	88	13	**28.0**	**0.58**	-PKAEFAE-------	0.9	1.8	1.2	2.3	2.9	2.9	1.7	2.2	0.5	0.4	0.0	0.2	0.6	0.5	0.4
248	PKAEFAEVSKLVTDL	123	26	**16.7**	**0.45**	P-AEFAEVS-LV--L	1.0	0.6	1.5	2.2	1.8	1.3	3.2	1.2	1.7	0.9	1.6	1.8	0.2	0.3	1.6
249	KAEFAEVSKLVTDLT	124	26	**29.4**	**0.59**	--E--EVSKLV--LT	0.0	0.4	1.3	0.8	0.3	2.5	1.5	3.0	2.0	2.7	3.0	0.8	0.6	2.3	1.2
250	AEFAEVSKLVTDLTK	135	19	**34.8**	**0.63**	-E--EVSKLV--LT-	0.2	1.0	0.6	0.3	2.2	1.4	3.2	2.2	2.9	2.9	0.7	0.9	2.8	1.6	0.0
251	EFAEVSKLVTDLTKV	113	22	**32.6**	**0.62**	---EVSKLV--LT--	0.8	0.3	0.2	2.2	1.0	3.1	2.0	2.7	3.0	0.5	0.8	2.5	1.2	0.2	0.2
252	FAEVSKLVTDLTKVH	78	17	**39.3**	**0.66**	--EVSKLVTDLT---	0.4	0.3	2.6	1.3	3.1	1.9	2.6	2.8	1.1	1.3	2.3	1.5	0.0	0.3	0.0
253	AEVSKLVTDLTKVHT	62	13	**44.9**	**0.69**	-EVSKLV-DLT----	0.0	2.3	1.2	2.6	1.5	2.5	2.4	0.5	1.0	2.5	1.2	0.0	0.0	0.0	0.0
254	EVSKLVTDLTKVHTE	84	12	**40.0**	**0.66**	EVSKLV-DLT----E	2.5	1.6	3.0	2.0	2.8	2.9	0.6	1.0	2.7	1.4	0.3	0.3	0.0	0.4	1.2
255	VSKLVTDLTKVHTEC	16	9	**11.6**	0.36	----------------	0.5	0.7	0.4	0.5	1.0	0.6	0.6	0.8	0.8	0.0	0.0	0.0	0.0	0.8	0.0
256	SKLVTDLTKVHTECC	5	11	3.3	0.14	----------------	0.2	0.0	0.3	0.3	0.3	0.0	0.1	0.2	0.0	0.4	0.0	0.1	0.0	0.1	0.1
257	KLVTDLTKVHTECCH	-3	8	2.3	0.10	----------------	0.0	0.0	0.0	0.1	0.0	0.0	0.0	0.0	0.1	0.0	0.0	0.0	0.1	0.1	0.0
258	LVTDLTKVHTECCHG	3	7	2.2	0.10	----------------	0.0	0.0	0.0	0.0	0.0	0.0	0.0	0.2	0.0	0.0	0.1	0.0	0.2	0.0	0.0
259	VTDLTKVHTECCHGD	3	7	1.9	0.08	----------------	0.0	0.0	0.0	0.1	0.0	0.0	0.0	0.0	0.0	0.2	0.0	0.2	0.0	0.0	0.1
260	TDLTKVHTECCHGDL	2	5	1.9	0.08	----------------	0.0	0.0	0.0	0.0	0.0	0.1	0.0	0.0	0.0	0.0	0.0	0.0	0.0	0.0	0.0
261	DLTKVHTECCHGDLL	1	10	2.7	0.12	----------------	0.0	0.0	0.0	0.0	0.0	0.0	0.0	0.0	0.0	0.0	0.0	0.0	0.0	0.0	0.0
262	LTKVHTECCHGDLLE	4	7	1.6	0.07	----------------	0.0	0.0	0.0	0.0	0.0	0.1	0.0	0.0	0.0	0.0	0.1	0.0	0.1	0.0	0.0
263	TKVHTECCHGDLLEC	9	11	3.1	0.13	----------------	0.4	0.0	0.5	0.0	0.4	0.1	0.1	0.5	0.0	0.4	0.1	0.3	0.1	0.0	0.2
264	KVHTECCHGDLLECA	2	9	1.9	0.08	----------------	0.0	0.0	0.0	0.0	0.0	0.0	0.0	0.0	0.0	0.0	0.0	0.0	0.0	0.0	0.0
265	VHTECCHGDLLECAD	12	5	1.4	0.07	----------------	0.2	0.1	0.0	0.2	0.2	0.1	0.0	0.0	0.2	0.2	0.4	0.4	0.2	0.1	0.0
266	HTECCHGDLLECADD	6	3	2.3	0.10	----------------	0.0	0.1	0.0	0.0	0.0	0.0	0.0	0.0	0.3	0.1	0.0	0.0	0.0	0.0	0.0
267	TECCHGDLLECADDR	7	3	1.7	0.08	----------------	0.0	0.1	0.0	0.0	0.0	0.0	0.0	0.2	0.0	0.1	0.0	0.0	0.0	0.0	0.0
268	ECCHGDLLECADDRA	8	3	1.8	0.08	----------------	0.1	0.0	0.0	0.0	0.0	0.0	0.1	0.2	0.0	0.3	0.0	0.0	0.0	0.3	0.0
269	CCHGDLLECADDRAD	5	4	1.5	0.07	----------------	0.0	0.0	0.0	0.0	0.0	0.0	0.0	0.3	0.0	0.0	0.0	0.0	0.1	0.0	0.0
270	CHGDLLECADDRADL	8	4	1.4	0.07	----------------	0.3	0.0	0.1	0.0	0.2	0.1	0.3	0.0	0.0	0.1	0.0	0.0	0.1	0.0	0.5
271	HGDLLECADDRADLA	9	3	2.3	0.10	----------------	0.0	0.0	0.0	0.2	0.3	0.4	0.0	0.0	0.0	0.0	0.1	0.0	0.0	0.0	0.0
272	GDLLECADDRADLAK	9	7	1.4	0.06	----------------	0.0	0.0	0.0	0.0	0.3	0.0	0.0	0.0	0.0	0.0	0.0	0.1	0.1	0.0	0.0
273	DLLECADDRADLAKY	11	4	1.9	0.08	----------------	0.2	0.2	0.3	0.4	0.0	0.0	0.0	0.1	0.0	0.0	0.4	0.2	0.3	0.0	0.2
274	LLECADDRADLAKYI	14	3	2.7	0.12	----------------	0.4	0.1	0.8	0.1	0.1	0.4	0.3	0.0	0.1	0.3	0.4	0.2	0.0	0.2	0.2
275	LECADDRADLAKYIC	12	4	**4.3**	0.17	----------------	0.0	0.5	0.1	0.0	0.0	0.3	0.0	0.0	0.4	0.7	0.0	0.0	0.4	0.3	0.2
276	ECADDRADLAKYICE	12	5	2.9	0.12	----------------	0.3	0.0	0.0	0.0	0.0	0.0	0.0	0.0	0.3	0.0	0.0	0.3	0.4	0.4	0.0
277	CADDRADLAKYICEN	17	4	**6.2**	0.23	----------------	0.0	0.0	0.3	0.1	0.0	0.0	0.5	0.7	0.0	0.0	0.6	0.6	0.0	1.2	0.7
278	ADDRADLAKYICENQ	19	3	**7.4**	0.27	----------------	0.0	0.1	0.1	0.0	0.0	0.7	0.5	0.0	0.0	0.4	0.6	0.0	1.2	0.6	0.2
279	DDRADLAKYICENQD	24	10	**10.4**	0.34	----------------	0.2	0.6	0.0	0.5	1.3	1.3	0.4	0.2	1.4	1.2	0.0	1.8	1.1	0.5	0.4
280	DRADLAKYICENQDS	20	5	**12.6**	0.38	----------------	0.2	0.0	0.0	0.6	0.9	0.0	0.0	1.1	0.9	0.0	1.5	0.8	0.0	0.2	0.0
281	RADLAKYICENQDSI	16	4	**10.3**	0.34	----------------	0.0	0.1	0.7	0.6	0.0	0.0	0.7	0.8	0.0	1.2	0.6	0.0	0.3	0.0	0.2

282	ADLAKYICENQDSIS	22	9	**5.6**	0.22	----------------	0.1	0.6	0.9	0.0	0.0	1.1	0.9	0.4	1.3	0.8	0.2	0.7	0.4	0.6	0.2
283	DLAKYICENQDSISS	32	11	**11.8**	0.37	----------------	0.9	1.7	0.0	0.0	1.4	1.4	0.1	1.4	1.1	0.4	0.5	0.7	0.3	0.3	0.1
284	LAKYICENQDSISSK	18	4	**9.6**	0.32	----------------	0.6	0.0	0.0	0.3	0.7	0.0	0.7	0.0	0.4	0.0	0.3	0.6	0.3	0.0	0.0
285	AKYICENQDSISSKL	12	3	**6.3**	0.24	----------------	0.0	0.0	0.1	0.3	0.0	0.5	0.0	0.0	0.1	0.5	0.7	0.5	0.1	0.0	0.5
286	KYICENQDSISSKLK	4	4	**19.7**	**0.49**	----------------	0.0	0.0	0.2	0.0	0.0	0.0	0.0	0.1	0.0	0.0	0.0	0.0	0.0	0.5	0.0
287	YICENQDSISSKLKE	25	10	**10.1**	0.33	----------------	0.0	0.0	0.0	0.0	0.0	0.0	0.1	0.9	1.5	1.1	0.2	0.0	1.0	0.0	0.7
288	ICENQDSISSKLKEC	27	4	**6.3**	0.24	----------------	0.1	0.1	0.2	0.0	0.0	0.2	0.7	1.2	1.0	0.4	0.1	0.7	0.0	0.9	0.2
289	CENQDSISSKLKECC	26	3	**11.5**	0.36	----------------	0.0	0.3	0.0	0.0	0.1	0.9	1.4	1.0	0.6	0.1	0.9	0.0	1.8	0.4	0.1
290	ENQDSISSKLKECCE	19	5	**4.9**	0.19	----------------	0.0	0.0	0.3	0.0	1.0	1.1	0.7	0.2	0.3	1.0	0.2	0.5	0.1	0.2	0.5
291	NQDSISSKLKECCEK	15	4	**8.5**	0.29	----------------	0.0	0.0	0.0	0.5	0.7	0.5	0.0	0.0	0.4	0.0	1.0	0.0	0.1	1.1	0.0
292	QDSISSKLKECCEKP	13	4	**11.9**	0.37	----------------	0.0	0.0	0.3	1.1	0.5	0.2	0.0	0.8	0.0	1.0	0.0	0.0	1.1	0.0	0.0
293	DSISSKLKECCEKPL	10	5	**9.5**	0.32	----------------	0.1	0.4	0.8	0.1	0.1	0.0	0.2	0.0	1.4	0.0	0.3	1.2	0.0	0.0	0.0
294	SISSKLKECCEKPLL	7	6	**9.8**	0.33	----------------	0.0	0.4	0.0	0.0	0.0	0.3	0.0	0.6	0.0	0.0	0.0	0.0	0.0	0.0	0.0
295	ISSKLKECCEKPLLE	6	5	**5.0**	0.20	----------------	0.6	0.1	0.0	0.0	0.2	0.0	0.7	0.2	0.0	0.4	0.1	0.0	0.1	0.0	0.0
296	SSKLKECCEKPLLEK	2	3	**10.3**	0.34	----------------	0.1	0.0	0.0	0.2	0.0	0.0	0.0	0.0	0.0	0.0	0.0	0.0	0.0	0.0	0.0
297	SKLKECCEKPLLEKS	-1	18	**9.2**	0.31	----------------	0.0	0.0	0.0	0.0	0.0	0.0	0.0	0.0	0.0	0.0	0.1	0.0	0.0	0.0	0.0
298	KLKECCEKPLLEKSH	-5	4	**10.2**	0.33	----------------	0.0	0.0	0.0	0.0	0.3	0.0	0.0	0.7	0.1	0.3	0.1	0.0	0.4	0.3	0.0
299	LKECCEKPLLEKSHC	11	32	**3.7**	0.16	----------------	1.1	0.4	0.0	0.7	0.8	0.0	0.7	1.1	1.0	1.0	1.3	0.9	0.8	0.3	0.7
300	KECCEKPLLEKSHCI	1	2	**5.8**	0.22	----------------	0.0	0.0	0.0	0.0	0.0	0.0	0.0	0.0	0.1	0.0	0.0	0.0	0.0	0.1	0.0
301	ECCEKPLLEKSHCIA	5	3	**4.5**	0.18	----------------	0.1	0.0	0.0	0.4	0.0	0.0	0.0	0.1	0.4	0.0	0.0	0.0	0.2	0.2	0.2
302	CCEKPLLEKSHCIAE	2	4	3.2	0.14	----------------	0.0	0.0	0.1	0.1	0.0	0.1	0.1	0.0	0.0	0.0	0.0	0.0	0.0	0.0	0.0
303	CEKPLLEKSHCIAEV	3	3	2.4	0.11	----------------	0.0	0.2	0.0	0.0	0.3	0.0	0.0	0.0	0.0	0.0	0.0	0.0	0.1	0.0	0.0
304	EKPLLEKSHCIAEVE	3	4	2.6	0.11	----------------	0.3	0.0	0.0	0.0	0.2	0.1	0.0	0.1	0.0	0.0	0.0	0.0	0.0	0.4	0.0
305	KPLLEKSHCIAEVEN	1	5	3.1	0.13	----------------	0.0	0.0	0.0	0.0	0.0	0.0	0.0	0.0	0.1	0.1	0.0	0.0	0.0	0.0	0.0
306	PLLEKSHCIAEVEND	7	4	**4.1**	0.17	----------------	0.0	0.2	0.3	0.3	0.0	0.1	0.0	0.2	0.7	0.1	0.0	0.4	0.0	0.4	0.1
307	LLEKSHCIAEVENDE	5	6	**3.7**	0.15	----------------	0.0	0.2	0.1	0.0	0.0	0.0	0.3	0.5	0.0	0.0	0.0	0.0	0.2	0.0	0.0
308	LEKSHCIAEVENDEM	3	3	1.2	0.05	----------------	0.0	0.0	0.0	0.0	0.0	0.0	0.0	0.2	0.0	0.0	0.0	0.0	0.0	0.0	0.0
309	EKSHCIAEVENDEMP	1	3	1.9	0.09	----------------	0.0	0.0	0.0	0.0	0.0	0.0	0.0	0.0	0.0	0.0	0.0	0.0	0.0	0.0	0.0
310	KSHCIAEVENDEMPA	58	23	**32.4**	**0.61**	-----------DEMPA	0.2	0.3	0.0	0.3	0.6	0.4	0.4	0.0	0.3	0.5	2.4	2.6	2.6	2.2	2.5
311	SHCIAEVENDEMPAD	44	20	**13.7**	**0.40**	----------DEMPAD	0.4	0.0	0.4	0.6	0.4	0.8	0.4	0.5	0.3	1.8	1.7	1.8	1.6	1.8	1.7
312	HCIAEVENDEMPADL	141	44	**25.3**	**0.55**	----------EMPAD-	0.0	0.0	0.0	0.0	0.0	0.0	0.2	0.0	1.0	1.9	1.8	1.5	1.8	1.7	0.9
313	CIAEVENDEMPADLP	271	106	**25.4**	**0.55**	--------DEMPADL-	0.0	0.0	0.0	0.2	0.0	0.0	0.1	1.2	2.0	1.1	1.7	1.9	2.1	1.8	1.0
314	IAEVENDEMPADLPS	317	80	**25.2**	**0.55**	--------EMPADLP-	0.0	0.2	0.1	0.1	0.0	0.0	0.9	2.1	1.1	1.7	1.9	2.4	1.8	1.1	0.1
315	AEVENDEMPADLPSL	349	88	**26.0**	**0.56**	------DEMPADLP--	0.0	0.1	0.3	0.4	0.0	1.1	2.3	1.1	1.9	1.8	2.5	2.2	1.5	0.0	0.1
316	EVENDEMPADLPSLA	400	117	**34.0**	**0.63**	----DEMPADLP-L-	0.1	0.1	0.4	0.5	1.0	2.8	1.2	2.4	2.3	3.1	2.9	2.0	0.7	1.0	0.4
317	VENDEMPADLPSLAA	349	98	**29.2**	**0.59**	----E-PADLP----	0.0	0.0	0.0	0.6	2.3	0.8	1.9	1.6	2.7	2.5	1.8	0.5	0.4	0.0	0.2
318	ENDEMPADLPSLAAD	304	85	**25.1**	**0.55**	---EMPADLP-----	0.0	0.0	0.9	2.0	1.1	1.9	1.5	2.5	2.1	1.8	0.3	0.5	0.0	0.1	0.1
319	NDEMPADLPSLAADF	354	98	**19.4**	**0.49**	-DE-PADLP-L--D-	0.0	1.1	2.3	0.9	1.9	1.6	2.7	2.5	2.2	0.5	1.1	0.7	0.5	1.0	0.7
320	DEMPADLPSLAADFV	516	54	**18.2**	**0.47**	-E---DL--L-ADFV	0.3	1.4	0.1	0.4	0.4	1.4	1.1	0.6	0.1	2.2	0.3	1.1	2.4	2.2	1.2
321	EMPADLPSLAADFVE	465	113	**36.8**	**0.64**	--------LAADFVE	0.3	0.0	0.3	0.4	0.7	0.8	0.6	0.5	3.1	1.2	2.0	3.1	3.0	1.7	2.1
322	MPADLPSLAADFVES	493	79	**69.7**	**0.77**	-------LAADFVE-	0.2	0.1	0.2	0.4	0.0	0.3	0.8	3.7	1.4	2.4	4.3	4.1	2.5	3.0	0.6
323	PADLPSLAADFVESK	553	48	**95.5**	**0.82**	------L-ADFVE--	0.2	0.0	0.1	0.0	0.0	0.4	2.5	0.6	1.6	5.1	5.0	2.8	3.8	0.7	0.6
324	ADLPSLAADFVESKD	528	56	**89.1**	**0.81**	-----L-ADFVE---	0.1	0.3	0.0	0.0	0.0	2.1	0.6	1.3	4.9	4.8	3.2	3.5	0.5	0.5	0.3
325	DLPSLAADFVESKDV	532	66	**87.6**	**0.81**	----L-ADFVE-K--	0.0	0.2	0.0	0.0	2.0	0.9	1.5	4.9	4.8	3.1	3.7	0.7	1.1	0.3	0.0
326	LPSLAADFVESKDVC	531	60	**76.8**	**0.79**	---L-ADFVE-K---	0.3	0.2	0.2	2.1	0.6	1.4	4.6	4.7	3.3	3.6	0.9	1.1	0.6	0.2	0.4
327	PSLAADFVESKDVCK	477	58	**79.3**	**0.80**	--L-ADFVESK----	0.0	0.0	1.8	0.7	1.6	4.5	4.4	3.2	3.7	1.0	1.2	0.7	0.0	0.0	0.0
328	SLAADFVESKDVCKN	434	68	**83.8**	**0.80**	-L-ADFVE-------	0.0	1.7	0.6	1.1	4.3	4.3	3.2	3.5	0.8	0.5	0.6	0.0	0.0	0.0	0.0

329	LAADFVESKDVCKNY	349	56	**79.5**	**0.80**	L-ADFVE-K------	1.5	0.5	1.2	3.7	4.2	3.3	3.6	0.5	1.0	0.7	0.0	0.0	0.0	0.0	0.0
330	AADFVESKDVCKNYA	159	24	**86.7**	**0.81**	--DFVE-KD------	0.0	0.0	3.5	3.6	2.8	3.3	0.5	3.3	1.4	0.0	0.0	0.0	0.0	0.0	0.0
331	ADFVESKDVCKNYAE	214	26	**70.2**	**0.78**	-DFVE-KD-------	0.3	3.8	3.9	3.4	3.1	0.4	3.7	1.6	0.5	0.3	0.0	0.3	0.5	0.4	0.9
332	DFVESKDVCKNYAEA	180	34	**71.8**	**0.78**	DFVE-KD--------	3.6	3.6	3.2	3.1	0.0	3.5	1.3	0.0	0.0	0.0	0.2	0.1	0.0	0.6	0.3
333	FVESKDVCKNYAEAK	13	5	**6.3**	0.24	---------------	0.8	0.4	1.1	0.4	0.0	0.7	0.9	0.6	0.0	0.3	0.8	0.1	1.4	0.2	0.1
334	VESKDVCKNYAEAKD	7	4	**6.8**	0.25	---------------	0.1	0.8	0.0	0.0	0.3	0.4	0.3	0.0	0.0	0.0	0.0	0.0	0.0	0.0	0.4
335	ESKDVCKNYAEAKDV	3	2	**6.7**	0.25	---------------	0.0	0.2	0.0	0.4	0.0	0.0	0.0	0.0	0.2	0.0	0.0	0.0	0.0	0.2	0.2
336	SKDVCKNYAEAKDVF	-1	5	**11.2**	0.35	---------------	0.1	0.0	0.0	0.0	0.0	0.0	0.0	0.0	0.0	0.0	0.1	0.0	0.0	0.0	0.0
337	KDVCKNYAEAKDVFL	1	4	**10.2**	0.33	---------------	0.0	0.0	0.0	0.0	0.0	0.0	0.0	0.1	0.0	0.0	0.0	0.0	0.0	0.0	0.0
338	DVCKNYAEAKDVFLG	7	5	**4.5**	0.18	---------------	0.0	0.1	0.0	0.0	0.0	0.1	0.0	0.7	0.0	0.0	0.1	0.0	0.5	0.0	0.0
339	VCKNYAEAKDVFLGM	7	4	**4.3**	0.18	---------------	0.3	0.0	0.0	0.0	0.3	0.1	0.8	0.3	0.0	0.5	0.3	0.0	0.6	0.0	0.2
340	CKNYAEAKDVFLGMF	5	5	**9.3**	0.31	---------------	0.0	0.0	0.0	0.1	0.0	0.0	0.0	0.0	0.2	0.3	0.7	0.5	0.0	0.0	0.1
341	KNYAEAKDVFLGMFL	9	2	**8.2**	0.29	---------------	0.0	0.0	0.1	0.0	0.0	0.2	0.0	0.3	0.0	0.5	0.5	0.0	0.0	0.3	0.3
342	NYAEAKDVFLGMFLY	19	3	**5.4**	0.21	---------------	0.0	0.4	0.1	1.1	0.0	0.0	0.2	0.0	0.6	0.2	0.0	0.0	0.6	0.3	0.0
343	YAEAKDVFLGMFLYE	25	6	**6.2**	0.23	---------------	0.0	0.2	1.0	0.3	0.0	0.5	0.3	0.7	0.5	0.0	0.0	0.7	0.2	0.2	0.6
344	AEAKDVFLGMFLYEY	27	10	3.4	0.14	---------------	0.0	0.6	0.0	0.0	0.6	0.0	0.1	0.2	0.0	0.1	0.1	0.0	0.4	0.6	0.0
345	EAKDVFLGMFLYEYA	27	4	**4.7**	0.19	---------------	0.9	0.0	0.1	0.7	0.0	0.1	0.2	0.0	0.0	0.4	0.5	0.2	1.1	0.3	0.0
346	AKDVFLGMFLYEYAR	12	5	**4.1**	0.17	---------------	0.2	0.0	0.6	0.1	0.0	0.2	0.0	0.0	0.3	0.1	0.2	0.5	0.0	0.0	0.0
347	KDVFLGMFLYEYARR	9	15	**5.7**	0.22	---------------	0.0	0.7	0.7	0.1	0.6	0.3	0.2	0.4	0.4	0.7	0.0	0.4	0.2	0.0	0.0
348	DVFLGMFLYEYARRH	5	5	2.9	0.12	---------------	0.1	0.0	0.0	0.0	0.0	0.1	0.0	0.0	0.0	0.3	0.2	0.0	0.0	0.0	0.0
349	VFLGMFLYEYARRHP	3	4	2.4	0.10	---------------	0.3	0.0	0.1	0.2	0.3	0.1	0.0	0.0	0.0	0.0	0.0	0.0	0.0	0.0	0.1
350	FLGMFLYEYARRHPD	4	38	3.0	0.13	---------------	0.0	0.1	0.0	0.2	0.2	0.0	0.3	0.0	0.2	0.0	0.0	0.0	0.0	0.0	0.2
351	LGMFLYEYARRHPDY	6	3	**5.2**	0.20	---------------	0.4	0.0	0.0	0.1	0.3	0.0	0.0	0.3	0.0	0.0	0.0	0.0	0.0	0.0	0.2
352	GMFLYEYARRHPDYS	3	13	**4.3**	0.18	---------------	0.0	0.0	0.1	0.0	0.0	0.3	0.0	0.0	0.0	0.0	0.0	0.0	0.2	0.1	0.0
353	MFLYEYARRHPDYSV	4	10	**3.9**	0.16	---------------	0.0	0.0	0.1	0.1	0.3	0.0	0.0	0.0	0.0	0.0	0.0	0.0	0.0	0.0	0.0
354	FLYEYARRHPDYSVV	7	5	**4.4**	0.18	---------------	0.4	0.3	0.0	0.5	0.2	0.0	0.0	0.0	0.0	0.0	0.2	0.1	0.2	0.1	0.0
355	LYEYARRHPDYSVVL	6	6	3.3	0.14	---------------	0.2	0.1	0.4	0.1	0.0	0.0	0.0	0.0	0.0	0.2	0.1	0.2	0.0	0.0	0.0
356	YEYARRHPDYSVVLL	4	6	**5.3**	0.21	---------------	0.0	0.0	0.0	0.0	0.0	0.0	0.0	0.0	0.2	0.0	0.0	0.0	0.0	0.0	0.0
357	EYARRHPDYSVVLLL	6	6	**3.6**	0.15	---------------	0.3	0.0	0.0	0.0	0.0	0.0	0.0	0.1	0.0	0.0	0.0	0.1	0.0	0.0	0.0
358	YARRHPDYSVVLLLR	1	3	**3.6**	0.15	---------------	0.0	0.0	0.0	0.0	0.0	0.0	0.0	0.0	0.0	0.0	0.0	0.0	0.0	0.1	0.0
359	ARRHPDYSVVLLLRL	1	2	1.5	0.07	---------------	0.0	0.0	0.0	0.0	0.0	0.0	0.0	0.0	0.0	0.0	0.0	0.0	0.0	0.0	0.0
360	RRHPDYSVVLLLRLA	1	16	2.5	0.11	---------------	0.0	0.0	0.0	0.1	0.0	0.0	0.0	0.0	0.0	0.0	0.0	0.0	0.0	0.0	0.0
361	RHPDYSVVLLLRLAK	-3	6	0.8	0.04	---------------	0.3	0.0	0.1	0.0	0.1	0.1	0.0	0.0	0.1	0.0	0.0	0.2	0.0	0.0	0.3
362	HPDYSVVLLLRLAKT	1	3	1.1	0.05	---------------	0.0	0.0	0.1	0.0	0.0	0.1	0.0	0.1	0.0	0.0	0.0	0.0	0.0	0.0	0.0
363	PDYSVVLLLRLAKTY	4	5	3.4	0.14	---------------	0.2	0.4	0.3	0.2	0.1	0.0	0.0	0.1	0.2	0.0	0.0	0.0	0.0	0.2	0.0
364	DYSVVLLLRLAKTYE	9	7	1.9	0.08	---------------	0.4	0.0	0.0	0.0	0.0	0.0	0.0	0.2	0.0	0.0	0.0	0.0	0.0	0.1	0.4
365	YSVVLLLRLAKTYET	11	5	2.6	0.11	---------------	0.2	0.4	0.0	0.1	0.0	0.0	0.0	0.2	0.0	0.0	0.0	0.3	0.4	0.8	0.0
366	SVVLLLRLAKTYETT	16	8	2.4	0.11	---------------	0.7	0.1	0.4	0.0	0.4	0.4	0.1	0.2	0.5	0.2	0.4	0.4	1.1	0.0	0.3
367	VVLLLRLAKTYETTL	12	4	1.7	0.08	---------------	0.0	0.0	0.0	0.0	0.0	0.0	0.1	0.0	0.0	0.0	0.1	0.6	0.0	0.0	0.0
368	VLLLRLAKTYETTLE	15	3	1.5	0.07	---------------	0.1	0.0	0.4	0.1	0.0	0.0	0.0	0.0	0.0	0.1	0.4	0.0	0.0	0.0	0.1
369	LLLRLAKTYETTLEK	19	4	**4.4**	0.18	---------------	0.5	0.2	0.3	0.1	0.8	0.0	0.0	0.2	0.1	0.9	0.4	0.2	0.0	0.6	0.3
370	LLRLAKTYETTLEKC	14	5	**4.0**	0.17	---------------	0.7	0.4	0.0	0.0	0.0	0.0	0.1	0.4	0.3	0.0	0.1	0.0	0.7	0.0	0.0
371	LRLAKTYETTLEKCC	10	7	**4.6**	0.18	---------------	0.5	0.0	0.6	0.0	0.0	0.1	0.2	0.3	0.0	0.0	0.1	0.5	0.0	0.0	0.1
372	RLAKTYETTLEKCCA	2	9	**5.8**	0.22	---------------	0.0	0.0	0.0	0.0	0.0	0.0	0.0	0.0	0.0	0.0	0.0	0.0	0.0	0.0	0.0
373	LAKTYETTLEKCCAA	11	17	2.2	0.10	---------------	0.2	0.0	0.0	0.0	0.2	0.6	0.0	0.0	0.0	0.6	0.0	0.4	0.2	0.0	0.1
374	AKTYETTLEKCCAAA	10	13	**4.6**	0.18	---------------	0.5	0.0	0.5	0.3	0.0	0.3	0.3	0.3	0.0	0.0	0.6	0.7	0.3	0.2	0.4
375	KTYETTLEKCCAAAD	6	4	2.4	0.10	---------------	0.0	0.2	0.1	0.5	0.2	0.0	0.0	0.0	0.0	0.0	0.1	0.0	0.0	0.0	0.2

376	TYETTLEKCCAAADP	14	3	2.5	0.11	---------------	0.0	0.2	0.3	0.0	0.0	0.0	0.9	0.0	0.0	0.0	0.0	0.1	0.0	0.1	0.0
377	YETTLEKCCAAADPH	7	10	2.2	0.10	---------------	0.2	0.1	0.2	0.1	0.0	0.6	0.0	0.0	0.2	0.0	0.2	0.3	0.1	0.0	0.0
378	ETTLEKCCAAADPHE	4	5	1.1	0.05	---------------	0.1	0.0	0.0	0.0	0.1	0.0	0.0	0.0	0.0	0.0	0.0	0.0	0.0	0.0	0.1
379	TTLEKCCAAADPHEC	8	10	**10.3**	0.34	---------------	0.0	0.0	0.0	0.2	0.0	0.0	0.0	0.0	0.3	0.1	0.3	0.2	0.0	0.4	0.2
380	TLEKCCAAADPHECY	17	16	**17.0**	**0.46**	---------------	0.3	0.2	0.6	0.0	0.6	0.0	0.1	0.6	0.5	0.8	0.6	0.0	0.8	0.5	0.1
381	LEKCCAAADPHECYA	16	12	**16.6**	**0.45**	-E---------E---	0.5	1.1	0.2	0.8	0.0	0.3	0.7	0.8	0.9	0.7	0.0	1.3	0.9	0.1	0.0
382	EKCCAAADPHECYAK	12	10	**21.3**	**0.51**	----------E----	0.9	0.0	0.6	0.0	0.2	0.7	0.0	0.9	0.8	0.0	0.0	1.0	0.1	0.0	0.0
383	KCCAAADPHECYAKV	2	4	**19.5**	**0.49**	---------------	0.0	0.0	0.0	0.1	0.0	0.0	0.0	0.0	0.0	0.0	0.0	0.0	0.0	0.0	0.0
384	CCAAADPHECYAKVF	14	6	**20.7**	**0.50**	--------E------	0.8	0.0	0.0	0.6	0.8	0.7	0.6	0.0	0.0	0.5	0.3	0.0	0.0	0.2	0.4
385	CAAADPHECYAKVFD	7	9	**3.8**	0.16	---------------	0.1	0.0	0.0	0.0	0.3	0.2	0.0	0.0	0.2	0.2	0.0	0.0	0.0	0.2	0.2
386	AAADPHECYAKVFDE	6	9	2.5	0.11	---------------	0.2	0.1	0.2	0.0	0.0	0.0	0.5	0.0	0.2	0.0	0.0	0.0	0.3	0.0	0.1
387	AADPHECYAKVFDEF	10	3	**5.6**	0.21	---------------	0.0	0.0	0.6	0.0	0.0	0.5	0.1	0.2	0.0	0.0	0.3	0.6	0.0	0.0	0.1
388	ADPHECYAKVFDEFK	90	14	**83.7**	**0.80**	---------VFDEFK	0.0	0.1	0.0	0.0	0.8	0.0	0.0	0.0	0.0	1.2	2.8	3.2	3.7	3.4	2.7
389	DPHECYAKVFDEFKP	188	29	**83.1**	**0.80**	---------FDEFKP	0.2	0.0	0.0	0.7	0.0	0.3	0.0	0.0	0.6	2.5	3.8	3.9	3.8	3.3	1.4
390	PHECYAKVFDEFKPL	292	46	**88.4**	**0.81**	--E-----FDEFKPL	0.6	0.0	1.0	0.2	0.4	0.3	0.0	0.9	2.9	4.6	4.6	4.5	3.1	2.0	1.0
391	HECYAKVFDEFKPLV	550	62	**109.2**	**0.84**	--------DEFKP--	0.1	0.5	0.0	0.2	0.1	0.0	0.0	0.9	4.4	4.8	4.6	3.7	3.1	0.5	0.8
392	ECYAKVFDEFKPLVE	621	44	**109.7**	**0.84**	-------DEFKP--E	0.2	0.0	0.0	0.2	0.0	0.0	0.5	3.9	5.2	5.3	4.2	2.7	0.3	0.5	2.0
393	CYAKVFDEFKPLVEE	629	49	**114.7**	**0.85**	------DEFKP--E-	0.2	0.1	0.2	0.0	0.3	0.6	4.0	5.3	5.4	4.6	3.3	0.4	0.8	2.0	0.4
394	YAKVFDEFKPLVEEP	645	40	**116.5**	**0.85**	-----DEFKP--E--	0.1	0.2	0.1	0.2	0.7	3.6	5.3	5.5	4.5	2.7	0.2	0.5	1.6	0.0	0.1
395	AKVFDEFKPLVEEPQ	612	39	**95.7**	**0.82**	----DEFKP--E---	0.0	0.0	0.1	0.4	2.6	4.6	4.7	3.9	2.6	0.3	0.3	1.3	0.2	0.0	0.0
396	KVFDEFKPLVEEPQN	615	35	**114.8**	**0.85**	---DEFKP--E----	0.0	0.2	0.6	2.5	4.9	5.4	4.5	3.3	0.1	0.6	2.1	0.3	0.2	0.0	0.0
397	VFDEFKPLVEEPQNL	620	35	**91.7**	**0.82**	--DEFKP--E-----	0.1	0.7	2.8	4.9	5.2	4.3	2.8	0.2	0.7	2.1	0.4	0.5	0.0	0.1	0.2
398	FDEFKPLVEEPQNLI	556	45	**90.2**	**0.82**	-DEFKP--E------	0.7	2.6	4.5	4.6	4.5	3.7	0.3	0.7	2.6	0.7	0.5	0.1	0.0	0.1	0.2
399	DEFKPLVEEPQNLIK	539	29	**94.7**	**0.82**	DEFKP-VEE------	2.5	4.3	4.6	4.4	4.3	0.5	1.4	3.8	1.2	0.8	0.3	0.0	0.3	0.2	0.1
400	EFKPLVEEPQNLIKQ	257	35	**70.1**	**0.78**	EFKP-VEEP------	3.4	3.4	3.1	3.5	0.4	1.7	3.9	1.6	1.5	0.2	0.2	0.0	0.0	0.0	0.0
401	FKPLVEEPQNLIKQN	38	8	**21.0**	**0.51**	---L-EEPQN-----	0.5	0.0	0.7	1.1	0.9	2.0	1.9	1.5	1.2	1.2	0.7	0.8	0.0	0.0	0.0
402	KPLVEEPQNLIKQNC	22	15	**28.1**	**0.58**	----EEP--------	0.0	0.2	1.0	0.7	0.0	0.0	1.1	0.7	1.0	0.1	0.0	0.0	0.0	0.0	0.0
403	PLVEEPQNLIKQNCE	57	10	**19.4**	**0.49**	-LVEEPQN-------	0.9	1.4	1.2	2.1	2.3	2.3	1.4	1.3	0.8	0.8	0.0	0.1	0.3	0.2	0.6
404	LVEEPQNLIKQNCEL	37	27	**9.4**	0.32	---------------	1.1	0.7	0.9	0.9	0.7	0.5	0.4	0.0	0.1	0.0	0.0	0.0	0.0	0.2	0.0
405	VEEPQNLIKQNCELF	19	11	1.8	0.08	---------------	0.2	0.3	0.2	0.0	0.1	0.0	0.4	0.3	0.0	0.1	0.2	0.0	0.6	0.0	0.1
406	EEPQNLIKQNCELFE	13	13	1.3	0.06	---------------	0.0	0.0	0.0	0.0	0.0	0.5	0.4	0.0	0.0	0.0	0.0	0.0	0.0	0.0	0.0
407	EPQNLIKQNCELFEQ	20	8	2.2	0.10	---------------	0.1	0.1	0.2	0.0	0.4	0.6	0.0	0.4	0.5	0.0	0.1	0.3	0.5	0.0	0.4
408	PQNLIKQNCELFEQL	14	26	2.5	0.11	---------------	0.0	0.0	0.0	0.0	0.0	0.0	0.0	0.0	0.0	0.0	0.2	0.1	0.0	0.0	0.0
409	QNLIKQNCELFEQLG	16	8	2.1	0.09	---------------	0.0	0.0	0.1	0.3	0.0	0.0	0.0	0.0	0.1	0.2	0.3	0.1	0.0	0.1	0.0
410	NLIKQNCELFEQLGE	21	3	3.0	0.13	---------------	0.1	0.3	0.5	0.0	0.0	0.0	0.0	0.4	0.6	0.5	0.4	0.0	0.4	0.0	0.1
411	LIKQNCELFEQLGEY	28	9	**3.8**	0.16	---------------	0.4	0.3	0.0	0.5	0.0	0.0	0.1	0.7	0.8	0.0	0.1	0.7	0.0	0.3	0.3
412	IKQNCELFEQLGEYK	20	10	3.2	0.14	---------------	0.2	0.0	0.0	0.0	0.0	0.5	0.3	0.4	0.5	0.3	0.2	0.0	0.6	0.0	0.0
413	KQNCELFEQLGEYKF	39	8	**20.1**	**0.50**	---------L-EY-F	0.0	0.0	0.0	0.0	0.8	0.8	0.5	0.6	0.9	1.7	0.0	1.8	1.5	0.5	1.6
414	QNCELFEQLGEYKFQ	100	22	**27.1**	**0.57**	----LFE-LGEY-FQ	0.0	0.0	0.0	0.9	1.2	1.9	2.0	0.3	1.9	1.9	2.7	1.3	0.9	2.3	1.4
415	NCELFEQLGEYKFQN	97	19	**26.7**	**0.57**	--ELFE-LGEY-FQ-	0.1	0.0	1.3	1.0	2.1	2.2	0.3	2.1	1.7	2.7	1.8	0.8	2.6	1.9	0.3
416	CELFEQLGEYKFQNA	99	26	**12.6**	0.38	---------------	0.0	1.0	1.2	1.6	1.9	0.8	1.9	1.7	2.2	1.9	1.2	2.3	1.5	1.0	0.5
417	ELFEQLGEYKFQNAL	158	27	**18.8**	**0.48**	-LFEQLGEYKFQ---	0.6	1.0	1.9	2.2	1.3	2.3	2.4	2.4	1.8	1.3	2.6	1.6	0.7	0.0	0.3
418	LFEQLGEYKFQNALL	122	34	**20.8**	**0.51**	LFE-LGEY-FQ----	1.1	2.0	2.3	0.7	2.0	2.0	2.5	1.9	0.8	2.5	1.7	0.9	0.2	0.5	0.0
419	FEQLGEYKFQNALLV	60	15	**8.5**	0.29	---------------	0.9	1.6	0.5	1.4	1.2	1.3	1.2	0.2	1.4	0.7	0.7	0.2	0.4	0.0	0.0
420	EQLGEYKFQNALLVR	24	6	2.7	0.12	---------------	0.6	0.4	0.5	0.1	0.4	0.5	0.0	0.4	0.5	0.4	0.1	0.2	0.2	0.2	0.0
421	QLGEYKFQNALLVRY	24	8	3.3	0.14	---------------	0.5	0.7	0.5	1.1	0.9	0.1	0.3	0.0	0.5	0.4	0.5	0.4	0.5	0.0	0.2
422	LGEYKFQNALLVRYT	8	7	2.9	0.13	---------------	0.0	0.0	0.2	0.0	0.0	0.0	0.0	0.0	0.0	0.0	0.0	0.0	0.0	0.0	0.0

423	GEYKFQNALLVRYTK	-3	8	3.1	0.13	---------------	0.0	0.0	0.0	0.0	0.2	0.0	0.0	0.0	0.0	0.2	0.1	0.0	0.0	0.0	0.0
424	EYKFQNALLVRYTKK	1	11	1.1	0.05	---------------	0.0	0.0	0.0	0.0	0.0	0.0	0.0	0.0	0.0	0.0	0.0	0.0	0.0	0.0	0.0
425	YKFQNALLVRYTKKV	-3	14	1.2	0.06	---------------	0.0	0.0	0.1	0.1	0.0	0.0	0.0	0.0	0.1	0.0	0.0	0.0	0.1	0.0	0.1
426	KFQNALLVRYTKKVP	-6	8	0.9	0.04	---------------	0.0	0.0	0.0	0.0	0.2	0.0	0.0	0.0	0.0	0.0	0.0	0.1	0.0	0.0	0.0
427	FQNALLVRYTKKVPQ	-3	4	2.3	0.10	---------------	0.0	0.0	0.0	0.0	0.1	0.0	0.0	0.0	0.0	0.0	0.0	0.0	0.0	0.0	0.1
428	QNALLVRYTKKVPQV	-6	6	1.3	0.06	---------------	0.0	0.0	0.0	0.0	0.0	0.0	0.0	0.1	0.0	0.2	0.3	0.0	0.1	0.0	0.0
429	NALLVRYTKKVPQVS	-5	9	2.2	0.10	---------------	0.0	0.0	0.0	0.0	0.0	0.2	0.1	0.0	0.0	0.2	0.0	0.2	0.0	0.2	0.0
430	ALLVRYTKKVPQVST	1	7	2.1	0.09	---------------	0.0	0.0	0.0	0.0	0.0	0.0	0.0	0.0	0.0	0.0	0.0	0.0	0.0	0.0	0.0
431	LLVRYTKKVPQVSTP	1	7	2.3	0.10	---------------	0.0	0.0	0.0	0.0	0.0	0.0	0.0	0.0	0.0	0.0	0.0	0.0	0.0	0.0	0.0
432	LVRYTKKVPQVSTPT	-4	7	1.4	0.06	---------------	0.0	0.0	0.0	0.0	0.0	0.0	0.0	0.0	0.0	0.0	0.0	0.0	0.0	0.1	0.0
433	VRYTKKVPQVSTPTL	-7	7	1.9	0.08	---------------	0.3	0.0	0.2	0.1	0.2	0.3	0.0	0.3	0.0	0.0	0.0	0.2	0.2	0.0	0.0
434	RYTKKVPQVSTPTLV	-10	6	1.7	0.08	---------------	0.3	0.1	0.0	0.4	0.6	0.3	0.3	0.0	0.0	0.0	0.3	0.0	0.0	0.0	0.2
435	YTKKVPQVSTPTLVE	-5	11	2.9	0.13	---------------	0.0	0.0	0.0	0.0	0.0	0.0	0.1	0.1	0.1	0.0	0.2	0.0	0.1	0.1	0.0
436	TKKVPQVSTPTLVEV	-4	8	**4.1**	0.17	---------------	0.0	0.0	0.0	0.0	0.0	0.0	0.0	0.0	0.1	0.2	0.0	0.1	0.1	0.0	0.0
437	KKVPQVSTPTLVEVS	-6	10	2.6	0.11	---------------	0.0	0.0	0.0	0.0	0.2	0.0	0.0	0.1	0.0	0.1	0.0	0.0	0.0	0.1	0.0
438	KVPQVSTPTLVEVSR	6	21	**3.6**	0.15	---------------	0.0	0.2	0.0	0.0	0.1	0.0	0.0	0.0	0.1	0.1	0.1	0.0	0.0	0.1	0.0
439	VPQVSTPTLVEVSRN	14	22	1.0	0.05	---------------	0.0	0.0	0.0	0.0	0.0	0.0	0.0	0.0	0.0	0.0	0.2	0.0	0.0	0.0	0.0
440	PQVSTPTLVEVSRNL	18	8	1.4	0.06	---------------	0.0	0.1	0.1	0.3	0.0	0.0	0.2	0.3	0.1	0.4	0.1	0.0	0.0	0.4	0.2
441	QVSTPTLVEVSRNLG	30	6	**5.9**	0.23	---------------	0.7	0.3	0.3	0.0	0.1	0.3	0.4	0.4	1.6	1.0	1.2	0.6	0.9	1.1	0.2
442	VSTPTLVEVSRNLGK	27	9	**15.3**	**0.43**	-------E-S-NLG-	0.0	0.0	0.0	0.0	0.1	0.3	0.5	1.8	0.9	1.2	0.8	1.2	1.5	1.2	0.0
443	STPTLVEVSRNLGKV	37	28	**15.9**	**0.44**	------EVSRNLG--	0.4	0.3	0.4	0.6	0.4	0.9	0.0	1.4	2.0	1.6	1.8	1.8	1.5	0.1	0.6
444	TPTLVEVSRNLGKVG	36	12	**20.0**	**0.50**	-----E-SRNLG---	0.0	0.0	0.1	0.2	0.3	1.8	0.6	1.6	1.4	1.5	1.9	1.3	0.0	0.2	0.0
445	PTLVEVSRNLGKVGS	36	8	**26.3**	**0.56**	----E-SRNLG----	0.0	0.0	0.0	0.0	2.0	1.0	2.0	1.7	1.9	1.9	1.2	0.0	0.2	0.0	0.0
446	TLVEVSRNLGKVGSK	12	13	**8.0**	0.28	---------------	0.0	0.2	0.2	0.7	0.6	0.0	0.5	0.7	0.0	0.5	0.0	0.0	0.0	0.0	0.0
447	LVEVSRNLGKVGSKC	8	7	**8.8**	0.30	---------------	0.4	0.2	0.0	0.0	0.0	0.0	0.0	0.0	0.0	0.0	0.2	0.0	0.3	0.0	0.0
448	VEVSRNLGKVGSKCC	5	8	**8.7**	0.30	---------------	0.0	0.0	0.0	0.0	0.0	0.0	0.0	0.0	0.0	0.0	0.0	0.2	0.0	0.0	0.0
449	EVSRNLGKVGSKCCK	-7	3	**8.9**	0.30	---------------	0.0	0.0	0.0	0.1	0.0	0.0	0.0	0.9	0.0	0.0	0.0	1.3	0.0	0.0	0.4
450	VSRNLGKVGSKCCKH	-13	4	**4.1**	0.17	---------------	0.0	0.0	0.2	0.0	0.0	0.1	0.6	0.0	0.2	0.0	0.8	0.1	0.3	0.8	0.0
451	SRNLGKVGSKCCKHP	-14	3	2.8	0.12	---------------	0.0	0.2	0.2	0.0	0.1	0.3	0.0	0.0	0.0	0.5	0.3	0.0	0.6	0.0	0.2
452	RNLGKVGSKCCKHPE	-14	3	**5.0**	0.20	---------------	0.5	0.1	0.0	0.2	1.0	0.0	0.3	0.0	0.9	0.1	0.2	0.8	0.4	0.2	0.0
453	NLGKVGSKCCKHPEA	-10	4	**6.5**	0.24	---------------	0.0	0.0	0.0	0.7	0.0	0.0	0.1	0.9	0.0	0.0	0.6	0.5	0.0	0.0	0.0
454	LGKVGSKCCKHPEAK	-11	4	**6.8**	0.25	---------------	0.0	0.1	0.8	0.0	0.0	0.0	0.3	0.0	0.0	0.5	0.0	0.0	0.0	0.0	0.4
455	GKVGSKCCKHPEAKR	-15	4	**4.3**	0.17	---------------	0.0	0.6	0.0	0.0	0.0	0.8	0.0	0.0	0.5	0.3	0.2	0.0	0.0	0.5	0.1
456	KVGSKCCKHPEAKRM	-15	5	2.7	0.12	---------------	0.6	0.0	0.0	0.0	0.4	0.0	0.2	0.3	0.3	0.0	0.0	0.0	0.4	0.0	0.0
457	VGSKCCKHPEAKRMP	-15	4	**4.1**	0.17	---------------	0.0	0.4	0.0	0.7	0.2	0.2	0.9	0.4	0.3	0.0	0.5	1.0	0.7	0.6	0.3
458	GSKCCKHPEAKRMPC	-13	4	**5.5**	0.21	---------------	0.0	0.0	0.5	0.0	0.0	0.5	0.0	0.0	0.0	0.0	0.7	0.6	0.2	0.1	0.0
459	SKCCKHPEAKRMPCA	-13	3	**8.7**	0.30	---------------	0.0	1.2	0.1	0.0	0.7	0.0	0.0	0.0	0.0	1.0	0.1	0.0	0.0	0.1	0.0
460	KCCKHPEAKRMPCAE	-11	3	**7.0**	0.26	---------------	0.7	0.2	0.1	0.6	0.1	0.0	0.0	0.1	0.7	0.7	0.0	0.3	0.0	0.0	0.0
461	CCKHPEAKRMPCAED	-5	4	**10.4**	0.34	---------------	0.0	0.2	0.0	0.5	0.3	0.0	0.0	1.1	0.4	0.0	0.3	0.0	0.3	0.0	0.0
462	CKHPEAKRMPCAEDY	-3	3	**9.3**	0.31	---------------	0.0	0.0	0.3	0.0	0.0	0.0	0.0	0.2	0.5	0.5	0.0	0.0	0.0	0.0	0.0
463	KHPEAKRMPCAEDYL	-2	2	**8.8**	0.30	---------------	0.0	0.1	0.3	0.0	0.0	0.0	0.2	0.0	0.3	0.0	0.2	0.0	0.0	0.1	0.1
464	HPEAKRMPCAEDYLS	4	4	**6.3**	0.24	---------------	0.0	0.0	0.0	0.0	0.0	0.0	0.0	0.0	0.2	0.0	0.5	0.0	0.2	0.0	0.0
465	PEAKRMPCAEDYLSV	7	3	**7.5**	0.27	---------------	0.0	0.9	0.0	0.0	0.0	0.0	0.0	0.1	0.0	0.4	0.0	0.4	0.1	0.1	0.0
466	EAKRMPCAEDYLSVV	8	3	**6.7**	0.25	---------------	0.9	0.0	0.0	0.0	0.0	0.0	0.0	0.0	0.3	0.0	0.0	0.3	0.0	0.1	0.0
467	AKRMPCAEDYLSVVL	5	3	**12.3**	0.38	---------------	0.0	0.0	0.0	0.0	0.0	0.0	0.0	0.0	0.0	0.1	0.4	0.0	0.2	0.1	0.1
468	KRMPCAEDYLSVVLN	8	3	**20.6**	**0.51**	---------------	0.0	0.0	0.0	0.0	0.0	0.1	1.2	0.1	0.3	0.9	0.3	0.5	0.5	0.6	0.2
469	RMPCAEDYLSVVLNQ	24	4	**11.6**	0.36	---------------	0.0	0.0	0.0	0.0	0.0	1.5	0.0	0.5	1.3	0.9	1.3	0.6	1.0	0.0	0.5

470	MPCAEDYLSVVLNQL	34	7	**16.2**	**0.45**	----E--LS-VL---	0.1	0.0	0.0	0.0	1.6	0.0	0.1	1.7	1.2	0.9	1.2	1.4	0.0	0.2	0.9
471	PCAEDYLSVVLNQLC	32	4	**11.1**	0.35	---------------	0.0	0.0	0.1	1.8	0.3	0.3	1.3	1.4	0.9	1.2	1.3	0.5	0.5	1.0	0.0
472	CAEDYLSVVLNQLCV	31	4	**9.6**	0.32	---------------	0.0	0.0	1.4	0.3	0.1	0.9	1.1	0.8	0.5	0.5	0.4	0.5	0.5	0.0	0.0
473	AEDYLSVVLNQLCVL	37	5	**13.6**	**0.40**	-E--LS-V--Q----	0.0	1.6	0.6	0.6	1.1	1.0	0.9	1.0	0.0	0.3	1.0	0.7	0.0	0.3	0.6
474	EDYLSVVLNQLCVLH	22	5	**14.9**	**0.42**	E--------------	1.3	0.2	0.6	0.7	0.7	0.5	0.7	0.1	0.1	0.7	0.3	0.0	0.1	0.2	0.0
475	DYLSVVLNQLCVLHE	14	3	**14.5**	**0.42**	---------------	0.2	0.1	0.2	0.8	0.0	0.3	0.0	0.3	0.4	0.0	0.0	0.0	0.1	0.0	0.5
476	YLSVVLNQLCVLHEK	11	4	**17.2**	**0.46**	-------------E-	0.0	0.2	0.7	0.0	0.0	0.0	0.2	0.2	0.0	0.0	0.1	0.0	0.0	1.6	0.0
477	LSVVLNQLCVLHEKT	11	3	**11.6**	0.36	---------------	0.5	0.4	0.1	0.0	0.0	0.1	0.2	0.0	0.0	0.0	0.0	0.0	1.3	0.0	0.0
478	SVVLNQLCVLHEKTP	7	3	**10.3**	0.34	---------------	0.0	0.2	0.0	0.0	0.0	0.2	0.2	0.0	0.1	0.1	0.0	0.0	0.0	0.0	0.0
479	VVLNQLCVLHEKTPV	9	12	**8.4**	0.29	---------------	0.5	0.7	0.6	0.5	0.5	0.2	0.1	0.7	0.3	0.0	0.0	0.0	0.4	0.3	0.1
480	VLNQLCVLHEKTPVS	7	3	**11.9**	0.37	---------------	0.1	0.2	0.0	0.0	0.0	0.0	0.1	0.1	0.0	1.2	0.0	0.0	0.0	0.0	0.0
481	LNQLCVLHEKTPVSD	8	3	**7.1**	0.26	---------------	0.3	0.0	0.1	0.1	0.0	0.3	0.1	0.0	1.0	0.0	0.0	0.0	0.2	0.0	0.0
482	NQLCVLHEKTPVSDR	3	3	**6.8**	0.25	---------------	0.0	0.0	0.0	0.0	0.0	0.0	0.0	0.0	0.0	0.0	0.0	0.0	0.0	0.0	0.0
483	QLCVLHEKTPVSDRV	6	4	**12.6**	0.38	---------------	0.0	0.3	0.0	0.3	0.3	0.0	0.0	0.0	0.0	0.0	0.3	0.0	0.3	0.0	0.0
484	LCVLHEKTPVSDRVT	9	5	**7.2**	0.26	---------------	0.4	0.0	0.3	0.5	0.0	1.3	0.0	0.4	0.0	0.5	0.4	0.2	0.0	0.0	0.1
485	CVLHEKTPVSDRVTK	2	2	**8.1**	0.28	---------------	0.0	0.2	0.0	0.0	0.0	0.0	0.0	0.0	0.2	0.0	0.1	0.0	0.3	0.0	0.0
486	VLHEKTPVSDRVTKC	2	3	**13.5**	**0.40**	---------------	0.0	0.1	0.0	0.0	0.0	0.0	0.0	0.0	0.1	0.2	0.0	0.3	0.2	0.0	0.1
487	LHEKTPVSDRVTKCC	1	3	**15.6**	**0.43**	---------------	0.0	0.0	0.0	0.0	0.0	0.0	0.0	0.0	0.0	0.0	0.0	0.0	0.0	0.0	0.0
488	HEKTPVSDRVTKCCT	-1	3	**14.3**	**0.41**	---------------	0.0	0.0	0.0	0.0	0.0	0.0	0.0	0.0	0.0	0.0	0.0	0.0	0.0	0.0	0.0
489	EKTPVSDRVTKCCTE	8	3	**11.3**	0.36	---------------	1.1	0.0	0.1	0.0	0.1	0.3	0.0	0.0	0.7	0.2	0.0	0.0	0.0	0.0	1.1
490	KTPVSDRVTKCCTES	4	3	**14.5**	**0.42**	---------------	0.0	0.2	0.0	0.5	0.5	0.4	0.0	0.6	0.6	0.0	0.3	0.1	0.0	0.0	0.2
491	TPVSDRVTKCCTESL	13	3	**4.3**	0.17	---------------	0.0	0.0	0.0	0.2	0.0	0.0	0.3	0.2	0.0	0.0	0.0	0.1	1.0	0.0	0.1
492	PVSDRVTKCCTESLV	10	4	**4.7**	0.19	---------------	0.0	0.1	0.2	0.1	0.0	0.3	0.1	0.0	0.0	0.0	0.2	0.8	0.2	0.0	0.0
493	VSDRVTKCCTESLVN	16	3	**4.0**	0.16	---------------	0.2	0.3	0.6	0.0	0.7	0.3	0.0	0.1	0.0	0.1	1.1	0.4	0.4	0.1	0.3
494	SDRVTKCCTESLVNR	12	3	**9.7**	0.32	---------------	0.2	0.7	0.0	0.5	0.5	0.0	0.0	0.0	0.3	1.5	0.3	0.3	0.7	0.6	0.0
495	DRVTKCCTESLVNRR	7	4	**16.6**	**0.45**	---------------	0.1	0.0	0.7	0.5	0.0	0.1	0.0	0.2	0.0	0.7	0.8	0.7	0.2	0.0	0.0
496	RVTKCCTESLVNRRP	1	2	**8.8**	0.30	---------------	0.0	0.0	0.0	0.0	0.1	0.0	0.1	0.0	0.0	0.0	0.0	0.0	0.0	0.0	0.0
497	VTKCCTESLVNRRPC	7	2	**8.6**	0.30	---------------	0.5	0.3	0.0	0.0	0.1	0.1	0.0	0.6	0.4	0.4	0.1	0.0	0.0	0.0	0.3
498	TKCCTESLVNRRPCF	5	4	**12.9**	0.39	---------------	0.2	0.0	0.0	0.0	0.0	0.0	0.4	0.5	0.0	0.0	0.0	0.0	0.0	0.0	0.5
499	KCCTESLVNRRPCFS	4	4	**14.7**	**0.42**	---------------	0.0	0.0	0.0	0.0	0.0	0.2	0.4	0.3	0.0	0.0	0.0	0.0	0.1	0.2	0.0
500	CCTESLVNRRPCFSA	16	4	**8.3**	0.29	---------------	0.4	0.0	0.5	1.5	0.9	0.7	0.7	0.3	0.0	0.0	0.0	0.1	0.6	0.3	0.2
501	CTESLVNRRPCFSAL	14	6	**7.2**	0.26	---------------	0.0	0.0	1.0	0.5	0.3	0.7	0.1	0.0	0.0	0.0	0.0	0.6	0.3	0.0	0.2
502	TESLVNRRPCFSALE	13	4	**3.9**	0.16	---------------	0.2	0.5	0.6	0.7	0.6	0.1	0.0	0.0	0.0	0.0	0.7	0.0	0.0	0.1	0.1
503	ESLVNRRPCFSALEV	15	3	**3.6**	0.15	---------------	0.4	0.2	0.7	0.3	0.0	0.3	0.0	0.0	0.0	0.8	0.1	0.0	0.1	0.5	0.0
504	SLVNRRPCFSALEVD	15	3	**8.4**	0.29	---------------	0.0	0.3	0.2	0.2	0.0	0.0	0.0	0.0	0.6	0.6	0.0	0.7	1.2	0.7	0.8
505	LVNRRPCFSALEVDE	11	11	3.3	0.14	---------------	0.0	0.1	0.4	0.0	0.0	0.0	0.0	0.3	0.0	0.0	0.5	0.0	0.2	0.5	0.0
506	VNRRPCFSALEVDET	15	4	3.3	0.14	---------------	0.0	0.0	0.0	0.1	0.0	0.0	0.3	0.0	0.0	0.8	0.5	0.4	0.4	0.0	0.0
507	NRRPCFSALEVDETY	411	57	**66.8**	**0.77**	--------LEVDETY	0.0	0.0	0.0	0.0	0.1	0.2	0.3	0.1	2.2	3.5	1.1	3.9	1.8	3.6	3.3
508	RRPCFSALEVDETYV	423	56	**51.4**	**0.72**	-------LEVDETY-	0.3	0.2	0.0	0.4	0.3	0.3	0.1	1.9	3.2	1.1	3.5	2.1	3.2	2.9	0.3
509	RPCFSALEVDETYVP	464	63	**58.0**	**0.74**	------LE-DETY--	0.0	0.0	0.0	0.3	0.1	0.0	1.8	3.2	0.8	3.7	1.6	3.4	2.7	0.0	0.3
510	PCFSALEVDETYVPK	546	39	**72.8**	**0.78**	-----LEVDETY---	0.5	0.0	0.5	0.3	0.2	1.9	3.6	1.0	4.4	2.2	4.1	3.1	0.1	0.3	0.3
511	CFSALEVDETYVPKE	496	62	**74.5**	**0.79**	----LE-DETY----	0.0	0.1	0.1	0.0	1.8	3.4	0.8	4.1	1.8	3.8	3.3	0.3	0.2	0.2	0.0
512	FSALEVDETYVPKEF	430	74	**75.1**	**0.79**	---LEVDETY-----	0.0	0.2	0.4	1.5	3.4	1.3	4.3	2.0	4.0	3.5	0.3	0.1	0.0	0.0	0.1
513	SALEVDETYVPKEFN	365	54	**50.3**	**0.71**	--LE-DETY------	0.0	0.1	1.7	2.5	0.6	3.5	1.4	3.3	2.8	0.3	0.7	0.0	0.0	0.1	0.0
514	ALEVDETYVPKEFNA	441	62	**62.2**	**0.75**	-LE-DETY-------	0.0	1.7	3.2	0.9	3.9	1.6	3.7	3.2	0.0	0.5	0.3	0.1	0.2	0.3	0.0
515	LEVDETYVPKEFNAE	270	70	**67.2**	**0.77**	LE-D-TY--------	1.9	2.5	0.7	3.8	0.9	3.5	2.9	0.0	0.0	0.0	0.0	0.0	0.0	0.0	0.0
516	EVDETYVPKEFNAET	70	18	**17.8**	**0.47**	EVDETYV--------	1.8	1.4	1.9	1.9	1.9	1.5	1.0	0.6	0.1	0.0	0.5	0.1	0.2	0.0	0.2

517	VDETYVPKEFNAETF	14	4	1.5	0.07	----------------	0.1	0.3	0.2	0.2	0.6	0.3	0.0	0.0	0.0	0.4	0.0	0.0	0.0	0.1	0.0
518	DETYVPKEFNAETFT	12	3	3.2	0.14	----------------	0.0	0.1	0.0	0.1	0.0	0.0	0.0	0.0	0.2	0.0	0.0	0.0	0.0	0.2	0.0
519	ETYVPKEFNAETFTF	17	2	**5.4**	0.21	----------------	0.0	0.0	0.1	0.0	0.0	0.0	0.0	0.4	0.0	0.0	0.0	0.0	0.5	0.0	0.3
520	TYVPKEFNAETFTFH	15	3	2.7	0.12	----------------	0.1	0.0	0.0	0.0	0.0	0.0	0.2	0.0	0.0	0.3	0.0	0.3	0.0	0.4	0.0
521	YVPKEFNAETFTFHA	16	5	**6.5**	0.24	----------------	0.0	0.1	0.0	0.0	0.4	0.5	0.0	0.0	0.4	0.0	1.0	0.2	0.8	0.0	0.0
522	VPKEFNAETFTFHAD	12	3	**5.9**	0.22	----------------	0.0	0.0	0.0	0.0	0.4	0.0	0.0	0.0	0.0	0.4	0.0	0.7	0.0	0.0	0.0
523	PKEFNAETFTFHADI	12	3	2.2	0.10	----------------	0.0	0.0	0.2	0.3	0.0	0.0	0.2	0.0	0.6	0.4	0.5	0.0	0.0	0.1	0.1
524	KEFNAETFTFHADIC	14	2	**3.5**	0.15	----------------	0.0	0.4	0.3	0.1	0.0	0.5	0.0	0.7	0.1	0.7	0.0	0.1	0.3	0.1	0.2
525	EFNAETFTFHADICT	18	5	**6.2**	0.23	----------------	0.4	0.5	0.0	0.2	0.8	0.0	1.0	0.1	0.5	0.0	0.0	0.0	0.2	0.0	0.0
526	FNAETFTFHADICTL	19	9	**4.4**	0.18	----------------	0.6	0.0	0.0	1.0	0.0	0.7	0.2	0.8	0.0	0.0	0.4	0.1	0.0	0.2	0.0
527	NAETFTFHADICTLS	12	15	**3.8**	0.16	----------------	0.0	0.0	0.7	0.0	0.1	0.0	0.0	0.0	0.0	0.1	0.2	0.0	0.0	0.0	0.0
528	AETFTFHADICTLSE	12	3	**5.3**	0.21	----------------	0.0	0.5	0.0	0.5	0.0	0.1	0.0	0.0	0.1	0.3	0.0	0.4	0.0	0.0	0.0
529	ETFTFHADICTLSEK	10	15	1.9	0.09	----------------	0.2	0.0	0.0	0.0	0.0	0.0	0.0	0.0	0.0	0.0	0.0	0.0	0.2	0.0	0.0
530	TFTFHADICTLSEKE	10	3	2.3	0.10	----------------	0.0	0.2	0.1	0.0	0.0	0.0	0.1	0.2	0.0	0.0	0.1	0.0	0.4	0.0	0.0
531	FTFHADICTLSEKER	13	4	2.2	0.10	----------------	0.1	0.0	0.5	0.0	0.0	0.5	0.4	0.1	0.2	0.3	0.2	0.4	0.0	0.5	0.2
532	TFHADICTLSEKERQ	14	8	**4.3**	0.18	----------------	0.0	0.3	0.0	0.0	0.0	0.0	0.0	0.0	0.2	0.4	0.2	0.2	0.7	0.2	0.3
533	FHADICTLSEKERQI	64	14	**36.2**	**0.64**	-------LSEKE-QI	0.2	0.0	0.2	0.2	0.3	0.0	0.0	1.8	2.4	2.4	2.5	2.5	0.8	2.2	2.3
534	HADICTLSEKERQIK	83	12	**38.9**	**0.66**	------LSEKE-QI-	0.0	0.0	0.2	0.4	0.0	0.5	2.2	2.5	2.6	2.5	2.8	0.8	1.4	2.3	0.1
535	ADICTLSEKERQIKK	75	14	**56.8**	**0.74**	-----LSEKE-QI--	0.1	0.2	0.0	0.0	0.0	2.4	2.8	3.0	2.7	3.0	0.4	1.0	2.3	0.0	0.0
536	DICTLSEKERQIKKQ	105	42	**59.8**	**0.75**	----LSEKE-QI---	0.0	0.0	0.0	0.0	1.7	2.7	2.8	2.7	2.8	0.0	1.4	2.3	0.0	0.0	0.0
537	ICTLSEKERQIKKQT	90	36	**55.1**	**0.73**	---LSEKE--I----	0.0	0.0	0.0	1.4	2.5	2.6	2.4	0.0	0.0	0.9	2.3	0.0	0.0	0.0	0.4
538	CTLSEKERQIKKQTA	83	16	**54.8**	**0.73**	--LSEKE-QI-----	0.0	0.1	2.0	2.8	0.0	2.7	0.0	0.0	1.1	2.5	0.0	0.0	0.0	0.7	0.1
539	TLSEKERQIKKQTAL	83	14	**46.6**	**0.70**	-LSEKE-QI------	0.1	1.9	2.8	2.8	2.6	2.9	0.0	1.7	2.6	0.0	0.0	0.5	0.9	0.0	0.1
540	LSEKERQIKKQTALV	80	33	**56.1**	**0.73**	LSEKE-QI-------	1.7	2.8	2.8	2.6	2.9	0.0	1.0	2.4	0.0	0.0	0.1	0.5	0.0	0.0	0.0
541	SEKERQIKKQTALVE	6	3	**7.1**	0.26	----------------	0.5	0.0	0.0	0.0	0.0	0.0	0.6	0.0	0.0	0.3	0.1	0.0	0.1	0.0	0.6
542	EKERQIKKQTALVEL	5	6	**6.4**	0.24	----------------	0.0	0.0	0.6	0.0	0.2	0.0	0.0	0.0	0.3	0.1	0.0	0.2	0.2	0.5	0.2
543	KERQIKKQTALVELV	1	4	**3.6**	0.15	----------------	0.0	0.0	0.0	0.0	0.0	0.0	0.0	0.0	0.0	0.0	0.0	0.0	0.0	0.0	0.0
544	ERQIKKQTALVELVK	6	4	**8.0**	0.28	----------------	0.5	0.0	0.1	0.6	0.0	0.0	0.1	0.0	0.0	0.2	0.2	0.6	0.2	0.3	0.0
545	RQIKKQTALVELVKH	-3	4	**7.5**	0.27	----------------	0.4	0.0	0.0	0.0	0.0	0.0	0.0	0.0	0.0	0.0	0.0	0.0	0.0	0.0	0.0
546	QIKKQTALVELVKHK	-7	8	**7.5**	0.27	----------------	0.2	0.0	0.6	0.9	0.2	0.0	0.0	0.0	0.0	0.0	0.2	0.0	0.7	1.0	0.8
547	IKKQTALVELVKHKP	-8	4	**4.9**	0.19	----------------	0.0	0.1	0.2	0.0	0.0	0.2	0.0	0.0	0.0	0.1	0.0	0.6	0.4	0.5	0.0
548	KKQTALVELVKHKPK	-15	10	**5.0**	0.20	----------------	0.7	1.0	0.2	0.1	0.3	0.0	0.0	0.1	0.0	0.1	0.8	0.6	1.0	0.0	0.4
549	KQTALVELVKHKPKA	-11	3	2.7	0.12	----------------	0.0	0.0	0.0	0.0	0.0	0.0	0.0	0.0	0.0	0.4	0.3	0.5	0.0	0.4	0.0
550	QTALVELVKHKPKAT	-8	5	**4.8**	0.19	----------------	0.2	0.0	0.1	0.0	0.0	0.0	0.1	0.0	0.6	0.6	0.5	0.4	1.1	0.2	0.0
551	TALVELVKHKPKATK	-10	8	2.7	0.12	----------------	0.0	0.0	0.0	0.2	0.0	0.0	0.0	0.3	0.6	0.2	0.3	0.6	0.0	0.0	0.4
552	ALVELVKHKPKATKE	-7	2	**3.6**	0.15	----------------	0.0	0.0	0.0	0.0	0.0	0.0	0.5	0.2	0.7	0.2	0.3	0.0	0.0	0.2	0.0
553	LVELVKHKPKATKEQ	-6	5	**5.1**	0.20	----------------	0.0	0.0	0.0	0.0	0.0	0.1	0.3	0.7	0.3	0.8	0.0	0.1	0.5	0.0	0.0
554	VELVKHKPKATKEQL	-8	4	**3.6**	0.15	----------------	0.0	0.0	0.0	0.0	0.5	0.3	0.7	0.3	0.3	0.3	0.0	0.2	0.0	0.1	0.0
555	ELVKHKPKATKEQLK	-10	3	**8.1**	0.29	----------------	0.6	0.0	0.0	0.1	0.5	0.0	0.1	0.3	0.0	0.0	0.0	0.0	0.2	0.0	0.0
556	LVKHKPKATKEQLKA	-6	7	**11.8**	0.37	----------------	0.0	0.0	0.0	0.1	0.0	0.0	0.0	0.0	0.0	0.0	0.0	0.0	0.0	0.0	0.0
557	VKHKPKATKEQLKAV	-12	3	**8.5**	0.30	----------------	0.0	0.2	0.1	0.3	0.1	0.0	0.0	0.0	0.0	0.0	0.2	0.0	0.1	0.2	0.2
558	KHKPKATKEQLKAVM	-11	9	**4.1**	0.17	----------------	0.0	0.1	0.0	0.0	0.0	0.0	0.0	0.0	1.0	0.0	0.0	0.0	0.0	0.0	0.0
559	HKPKATKEQLKAVMD	-9	7	**6.7**	0.25	----------------	0.7	0.6	0.0	0.9	0.0	0.0	0.0	0.0	0.0	0.0	0.0	0.0	0.3	0.0	0.0
560	KPKATKEQLKAVMDD	1	10	**13.5**	**0.40**	----------------	0.0	0.0	0.0	0.0	0.0	0.0	0.0	0.0	0.0	0.0	0.0	0.0	0.0	0.0	0.0
561	PKATKEQLKAVMDDF	5	11	**10.7**	0.35	----------------	0.0	0.0	0.0	0.0	0.2	0.0	0.1	0.0	0.4	0.0	0.0	0.3	0.0	0.0	0.2
562	KATKEQLKAVMDDFA	6	15	**12.3**	0.38	----------------	0.0	0.0	0.2	0.0	0.0	0.0	0.2	0.0	0.0	0.0	0.0	0.0	0.0	0.1	0.0
563	ATKEQLKAVMDDFAA	63	28	**14.0**	**0.41**	-TKEQLKA--DDF--	0.4	1.4	1.2	0.0	1.3	1.3	1.5	1.8	0.0	0.4	1.9	1.5	1.0	0.4	0.1

564	TKEQLKAVMDDFAAF	38	12	**11.8**	0.37	----------------	0.8	0.6	1.9	0.6	0.8	1.0	1.4	0.0	0.4	1.5	1.3	0.8	0.0	0.0	0.5
565	KEQLKAVMDDFAAFV	26	15	**9.6**	0.32	----------------	0.6	0.0	0.0	0.7	0.7	1.1	0.2	0.4	1.3	1.1	0.7	0.0	0.0	0.4	0.4
566	EQLKAVMDDFAAFVE	24	7	**4.8**	0.19	----------------	1.1	0.3	0.8	0.0	0.4	0.2	0.3	0.5	0.4	0.8	0.0	0.0	0.8	0.1	0.1
567	QLKAVMDDFAAFVEK	9	4	**5.3**	0.21	----------------	0.0	0.1	0.0	0.0	0.2	0.0	0.2	0.3	0.2	0.0	0.0	0.5	0.0	0.7	0.0
568	LKAVMDDFAAFVEKC	10	4	**11.4**	0.36	----------------	0.5	0.0	0.0	0.0	0.0	0.6	0.4	0.4	0.0	0.0	0.4	0.0	1.3	0.0	0.1
569	KAVMDDFAAFVEKCC	3	3	**13.1**	0.39	----------------	0.0	0.0	0.0	0.0	0.2	0.3	0.2	0.0	0.0	0.3	0.0	0.0	0.0	0.0	0.0
570	AVMDDFAAFVEKCCK	5	4	**7.5**	0.27	----------------	0.0	0.0	0.0	0.3	0.0	0.1	0.0	0.0	0.0	0.1	0.0	0.0	0.0	0.0	0.0
571	VMDDFAAFVEKCCKA	2	5	**9.9**	0.33	----------------	0.0	0.0	0.1	0.2	0.0	0.0	0.0	0.0	0.0	0.0	0.0	0.0	0.0	0.0	0.0
572	MDDFAAFVEKCCKAD	4	4	**8.7**	0.30	----------------	0.1	0.1	0.3	0.4	0.0	0.0	0.0	0.1	0.0	0.0	0.0	0.0	0.0	0.0	0.0
573	DDFAAFVEKCCKADD	7	6	**8.5**	0.30	----------------	0.4	0.2	0.3	0.0	0.0	0.0	0.3	0.0	0.0	0.0	0.0	0.0	0.0	0.3	0.0
574	DFAAFVEKCCKADDK	1	3	**11.6**	0.36	----------------	0.0	0.1	0.0	0.0	0.1	0.0	0.0	0.0	0.0	0.0	0.0	0.0	0.0	0.0	0.0
575	FAAFVEKCCKADDKE	4	3	**10.8**	0.35	----------------	0.0	0.0	0.0	0.2	0.2	0.0	0.0	0.0	0.0	0.0	0.2	0.0	0.0	0.0	0.0
576	AAFVEKCCKADDKET	2	5	**9.0**	0.31	----------------	0.0	0.0	0.1	0.2	0.0	0.0	0.0	0.0	0.0	0.0	0.0	0.0	0.0	0.0	0.0
577	AFVEKCCKADDKETC	6	5	**8.6**	0.30	----------------	0.0	0.2	0.0	0.0	0.0	0.1	0.0	0.0	0.0	0.5	0.4	0.0	0.0	0.2	0.2
578	FVEKCCKADDKETCF	9	7	**11.2**	0.36	----------------	0.1	0.0	0.9	0.0	0.0	0.0	0.0	0.1	0.6	0.5	0.0	0.0	0.2	0.2	0.2
579	VEKCCKADDKETCFA	21	16	**14.8**	**0.42**	-------DD---C--	0.2	0.9	0.0	0.9	0.0	0.0	0.4	0.0	1.2	0.0	1.0	1.0	1.0	0.4	0.4
580	EKCCKADDKETCFAE	78	38	**16.1**	**0.44**	-----ADD-ETC--E	0.2	0.0	0.8	0.5	0.0	1.1	1.4	1.8	0.0	1.1	1.7	1.6	1.0	0.7	1.5
581	KCCKADDKETCFAEE	60	30	**16.1**	**0.44**	----ADD-ETC--EE	0.0	0.9	0.0	0.0	1.2	1.6	0.0	0.0	1.2	1.8	1.6	0.8	0.7	1.6	1.2
582	CCKADDKETCFAEEG	73	36	**15.5**	**0.43**	C---DD-ETC--EE-	1.0	0.1	0.0	0.9	1.6	1.9	0.0	1.2	1.8	1.5	0.9	0.6	1.7	1.1	0.0
583	CKADDKETCFAEEGK	40	23	**10.9**	0.35	----------------	0.0	0.0	0.7	1.0	1.1	0.0	0.9	1.0	0.4	0.5	0.6	1.1	0.9	0.0	0.0
584	KADDKETCFAEEGKK	11	10	**15.7**	**0.44**	----------E----	0.0	0.4	0.7	0.0	0.0	0.0	0.0	0.5	0.5	0.5	0.0	0.0	0.0	0.0	0.0
585	ADDKETCFAEEGKKL	18	11	**13.3**	**0.40**	---------E-----	0.0	0.6	0.8	0.0	0.8	0.8	0.2	0.5	0.3	0.0	0.9	0.1	0.0	0.0	0.1
586	DDKETCFAEEGKKLV	9	5	**11.8**	0.37	----------------	0.0	0.6	0.0	0.0	0.5	0.2	0.6	0.7	0.0	0.0	0.1	0.0	0.0	0.2	0.0
587	DKETCFAEEGKKLVA	5	4	**12.1**	0.37	----------------	0.0	0.0	0.0	0.3	0.0	0.4	0.0	0.0	0.0	0.0	0.0	0.0	0.4	0.0	0.0
588	KETCFAEEGKKLVAA	1	7	**11.2**	0.36	----------------	0.0	0.0	0.0	0.0	0.0	0.0	0.0	0.0	0.0	0.0	0.0	0.0	0.0	0.0	0.0
589	ETCFAEEGKKLVAAS	6	4	**13.1**	0.39	----------------	0.0	0.3	0.2	0.4	0.2	0.7	0.0	0.0	0.0	0.0	0.4	0.2	0.0	0.0	0.3
590	TCFAEEGKKLVAASQ	1	4	**7.9**	0.28	----------------	0.0	0.0	0.0	0.0	0.0	0.0	0.0	0.0	0.0	0.0	0.0	0.0	0.0	0.0	0.0
591	CFAEEGKKLVAASQA	1	5	**12.5**	0.38	----------------	0.0	0.0	0.0	0.0	0.0	0.0	0.0	0.0	0.0	0.0	0.1	0.0	0.0	0.1	0.0
592	FAEEGKKLVAASQAA	2	5	**10.0**	0.33	----------------	0.0	0.0	0.0	0.0	0.0	0.0	0.0	0.3	0.2	0.1	0.0	0.0	0.0	0.0	0.0
593	AEEGKKLVAASQAAL	-1	6	**7.3**	0.27	----------------	0.0	0.0	0.0	0.0	0.0	0.0	0.0	0.0	0.0	0.0	0.0	0.0	0.0	0.0	0.1
594	EEGKKLVAASQAALG	1	5	**7.5**	0.27	----------------	0.0	0.0	0.0	0.0	0.0	0.0	0.1	0.0	0.0	0.0	0.0	0.0	0.0	0.0	0.0
595	EGKKLVAASQAALGL	-4	5	**12.0**	0.37	----------------	0.0	0.2	0.0	0.0	0.0	0.0	0.0	0.0	0.0	0.1	0.2	0.0	0.0	0.4	0.0
